# CAR-T-Cell Therapy for Systemic Lupus Erythematosus: A Comprehensive Overview

**DOI:** 10.3390/ijms251910511

**Published:** 2024-09-29

**Authors:** Haneen M. Abdalhadi, Walter W. Chatham, Fatima K. Alduraibi

**Affiliations:** 1 Department of Medicine, Division of Clinical Immunology and Rheumatology, University of Alabama at Birmingham, Birmingham, AL 35294, USA; haneen91@live.com; 2 Department of Medicine, Division of Clinical Immunology and Rheumatology, University of Nevada, Las Vegas, NV 89102, USA; w.chatham@unlv.edu; 3 Department of Medicine, Division of Clinical Immunology and Rheumatology, Harvard Teaching Hospital, Boston, MA 02215, USA; 4 Department of Medicine, Division of Clinical Immunology and Rheumatology, King Faisal Specialist Hospital and Research Center, Riyadh 11211, Saudi Arabia

**Keywords:** systemic lupus erythematosus, B cells, CAR-T-cell therapy

## Abstract

Systemic lupus erythematosus (SLE) is a complex autoimmune disorder characterized by the production of autoreactive B and T cells and cytokines, leading to chronic inflammation affecting multiple organs. SLE is associated with significant complications that substantially increase morbidity and mortality. Given its complex pathogenesis, conventional treatments for SLE often have significant side effects and limited efficacy, necessitating the exploration of novel therapeutic strategies. One promising approach is the use of chimeric antigen receptor (CAR)-T-cell therapy, which has shown remarkable success in treating refractory hematological malignancies. This review provides a comprehensive analysis of the current use of CAR-T-cell therapy in SLE.

## 1. Introduction

Systemic lupus erythematosus (SLE), a heterogeneous systemic autoimmune disorder, has an overall prevalence of 0.1% and primarily affects young females between the ages of 15 and 44 years [1]. SLE is more prevalent in non-Caucasian populations, especially African Americans and Native-American/Hispanics, in whom SLE morbidity and mortality rates tend to be higher [1,2,3]. The key characteristic of SLE pathogenesis is the production of autoantibodies against nuclear antigens, which results from defective apoptotic clearance and excessive neutrophil extracellular traps (NETs) [1]. Dysregulated innate and adaptive immune responses, especially excessive production of type I interferons, shift toward T helper 17 (Th17) cells over regulatory T (Treg) cells, and autoreactive B cells play an important role in the development of SLE [1].

SLE is a multiorgan disorder with a wide spectrum of organ involvement and disease severity. Symptoms vary from mild, including arthritis, fatigue, pleurisy, lymphadenopathy and skin disorders, to more severe complications, such as lupus nephritis, central nervous system involvement, and vasculitis. These severe complications occur in 30–60% of SLE patients and are associated with a poor prognosis [1,4]. Various therapeutic approaches have been utilized in the management of SLE, with varying degrees of success. In the past decade, belimumab (2011), anifrolumab (2021), and voclosporin (2021) have received Food and Drug Administration (FDA) approval for use in the treatment of lupus [5]. While these advancements have helped greater numbers of patients achieve redefined goals of low disease activity and remission as well as preserving organ function, cures remain elusive thereby necessitating lifelong immunosuppressive therapy.

Chimeric antigen receptor (CAR)-T-cell therapy is an innovative therapy that has revolutionized the treatment of B-cell hematological malignancies [6]. Recently, researchers have investigated the application of CAR-T-cell therapy in the treatment of autoimmune diseases, particularly SLE. While achieving a cure remains difficult, a single administration of CAR-T-cell therapy has enabled drug-free clinical and serological remission in several patients with severe refractory disease [7,8,9,10,11,12]. In this review, recent evidence highlighting the role of CAR-T cells in treating SLE is provided in the context of other current and investigational B cell directed therapies.

## 2. Implementation of B-Cell-Targeted Therapies in Lupus

B cells are pivotal in the immune system’s defense against pathogens through several mechanisms, including antibody production, antigen presentation, T-cell regulation and differentiation, and cytokine production [13]. Each B-cell is equipped with a unique antigen receptor known as the B-cell receptor (BCR). Upon BCR recognition of an antigen and subsequent B-cell activation, activated B cells undergo proliferation and differentiation, ultimately secreting specific antibodies from one of the following five classes: IgM, IgD, IgG, IgA, or IgE [14].

Autoreactive B cells, which mistakenly recognize host antigens, undergo strict regulation during early developmental stages in the bone marrow (central tolerance) and later during maturation in secondary lymphoid organs such as the spleen and lymph nodes (peripheral tolerance). A breach in central tolerance mechanisms contributes to the development of autoimmune diseases and some immunodeficiency disorders [14].

**Figure 1:**
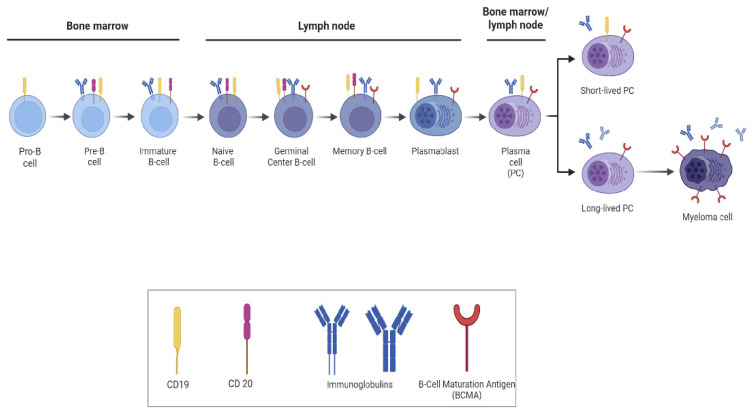
B-cell development and differentiation in the bone marrow and lymph nodes.

Furthermore, specific markers, including CD19, CD20, CD21, CD24, CD27, IgM, and IgD, can be used to identify the B-cell population in peripheral blood [15]. The levels of cell surface markers present on B cells change throughout the B-cell maturation stages. For example, CD19 remains consistently expressed on B cells from the initial stages of maturation, such as the pro-B-cell stage, all the way to their final differentiation into plasma cells. Conversely, CD20 is not present on either pro-B cells or plasma cells. These differences in antigen expression may impact therapeutic strategies and responses to treatment [13] (Figure 1).

Interestingly, B cells play a crucial role in the pathogenesis of SLE, and B-cell-targeted therapies have shown promising results in the management of SLE [16]. Compared with B-cell-depleted mice, MRL lpr/lpr mice with an intact B-cell population exhibit an exacerbation and progression of lupus-like symptoms, including increased severity of glomerulonephritis, vasculitis, and interstitial nephritis [17].

Additionally, B-cell targeted therapy for the management of SLE involves two main pathways: the inhibition of B-cell activation through the blockade of B-cell activating factor (BAFF), also termed B-lymphocyte stimulator (BLyS) and/or A-proliferation-inducing ligand (APRIL), and the depletion of B cells through the use of monoclonal antibodies (mAbs) against cell-surface antigens such as CD19, CD20, or CD22 [5,18,19].

Rituximab is a mAb that targets circulating mature B cells expressing the CD20 antigen. Rituximab induces B-cell depletion through antibody-dependent cellular cytotoxicity, complement-dependent cytotoxicity, and the induction of apoptosis [20]. Tissue-resident B cells and B cells lacking the CD20 antigen, including pro-B cells, plasma cells, and plasmablasts, evade antibody-mediated targeting by rituximab; therefore, rituximab is associated with a high risk of incomplete depletion of autoreactive B cells and resistance to therapy [21]. For example, B cells are still present in synovial biopsy samples from rheumatoid arthritis patients [22], tonsil samples from SLE patients [23], and abdominal lymph node samples from kidney transplant patients [24] who have been treated with rituximab, despite peripheral B-cell depletion.

The primary endpoints of two large randomized controlled clinical trials (RCTs) of rituximab use in the treatment of nonrenal (EXPLORER) and renal (LUNAR) manifestations of SLE were not met [25,26]. The lack of success in these trials was thought to be due to the trial design and the heavy background immunosuppression in the control group. The degree of B-cell depletion varies among patients, and those who experience more profound and sustained B-cell depletion tend to have better clinical outcomes [5].

Ocrelizumab is another anti-CD20 mAb that is used to treat lupus nephritis. A phase III randomized clinical trial (BELONG) demonstrated a numerical but not statistically significant improvement in renal outcomes in patients with class III/IV lupus nephritis treated with ocrelizumab [27]. The trial was terminated due to serious infections when ocrelizumab was combined with background mycophenolate (MMF) therapy.

Obinutuzumab, a fully humanized mAb against CD20, has shown efficacy in the treatment of patients with renal and nonrenal SLE who show no response to second-line rituximab [28]. In a recent randomized controlled trial, patients with proliferative lupus nephritis who received obinuzumab in addition to background MMF and prednisone therapy achieved an improved renal response compared with patients who received control treatment, and no safety signals were reported [19].

Another unique target for B-cell modulation is CD22, a B-lymphocyte-restricted adhesion molecule that when ligated downregulates BCR signaling. Epratuzumab, a recombinant humanized anti-CD22 mAb, initially led to reduced disease activity in an open-label RCT of 14 patients with lupus [29] but failed to lead to increased response rates compared with placebo in two phase III RCTs (EMBODY 1 and EMBODY 2) involving patients with moderately to severely active SLE [30].

Belimumab was the first FDA-approved biologic for the treatment of SLE [31]. Belimumab works by inhibiting BAFF, which is essential for the survival of B cells [32]. The BLISS-52 and BLISS-76 phase III trials demonstrated the efficacy of belimumab in reducing disease activity and flare rates in lupus patients, especially in the mucocutaneous and musculoskeletal domains [18,33]. Recently, the addition of belimumab to standard therapy for the management of lupus nephritis resulted in an increased renal response [34].

Tabalumab is another anti-BAFF mAb that has increased the SLE Responder Index (SRI) in ILLUMINATE, a phase III trial. However, because the secondary endpoints of the trial (time to severe flare, corticosteroid-sparing, and fatigue reduction) could not be achieved, the development of the medication was halted by the manufacturing company [35].

Another selective inhibitor of BAFF is blisibimod. Phase II (PEARL-SC) and III clinical trials of blisibimod did not meet their primary endpoint (SRI-6). However, blisibimod treatment led to improvements in patient-reported fatigue and disease activity [36,37,38].

The dual inhibition of APRIL and BAFF can be achieved by atacicept, which is a transmembrane activator and calcium-modulating cyclophilin ligand interactor (TACI-Ig) fusion protein [5]. An initial 52-week RCT revealed a reduced flare rate and longer time to first flare, with atacicept administered at 150 mg twice weekly but not at 75 mg twice weekly. Recruitment for the group receiving the higher dose of atacicept was halted owing to two deaths in that group [39]. However, a subsequent 24-week phase IIb study revealed an increased response rate (SRI-4) and a reduced flare rate among patients with high disease activity when both 150 mg and 75 mg twice-weekly dosages were used compared with the control treatment [40]. A long-term extension with a median treatment of 83.8 weeks demonstrated durable efficacy and no safety signal [41].

Given the promising results of B-cell targeted therapies in the management of SLE, the use of CAR-T cells against B-cell antigens, especially CD19, in SLE is thriving. Recently, increasing evidence has highlighted the potential of CD19- and BCMA-CD19-targeted CAR-T-cell therapy in the treatment of refractory autoimmune diseases, including SLE, systemic sclerosis (SSc), anti-synthetase syndrome, and multiple sclerosis [42].

## 3. Principles of B-Cell Depletion via CAR-T-Cell Therapy

Despite substantial advancements in the management of SLE, lifelong immunosuppressive and cytotoxic therapies remain imperative to maintain low disease activity or remission. Initial efforts to reset the immune system involved the utilization of autologous hematopoietic stem cell transplantation (HSCT) in patients with refractory lupus. A remission rate of 66% was reported in 53 SLE patients who underwent HSCT in Europe and Asia [43]. However, a relapse rate of 32% was documented among those who achieved remission, and the addition of steroids and other immunosuppressive therapies was needed. Furthermore, serious adverse events, such as infections, sepsis, the emergence of new immune events, and death, have also been reported [43,44].

CAR-T cells are genetically engineered T cells that have achieved substantial recognition within the past few years because of their demonstrated efficacy in treating small series of patients with autoimmune diseases [45]. The key components of CARs include an extracellular domain for ligand binding, a spacer domain, a transmembrane domain, and one or more cytoplasmic domains [46]. Single-chain variable fragments (scFvs) are the most utilized ligand-binding domains. The functionality of scFvs depends on their affinity, avidity, aggregation, and flexibility. The modulation of scFv affinity is a critical factor in increasing the specificity of CARs while minimizing off-tumor cytotoxic side effects [46]. For example, CARs with low-affinity scFv sequences demonstrated selective cytotoxicity toward highly expressing ErbB2 cells, whereas high-affinity variants did not [47]. Similarly, CARs with lower-affinity scFv sequences exhibited greater therapeutic efficacy in mice than CARs with high-affinity variants did, which was attributed to the ability of these low-affinity scFv CARs to discriminate between tumor tissue and normal tissues on the basis of antigen density [48]. The spacer domain is the connecting link between the scFv and the transmembrane domain. The spacer domain can be based on either IgG or a non-IgG marker, such as CD8 or CD28. The transmembrane domain within CAR structures relays ligand recognition signals to the intracellular cytoplasmic domain, which, in advanced generations of CARs, harbors costimulatory receptors (typically CD28, 4-1BB or both) that contribute to increased T-cell differentiation and activation-induced cell death [46].

**Figure 2:**
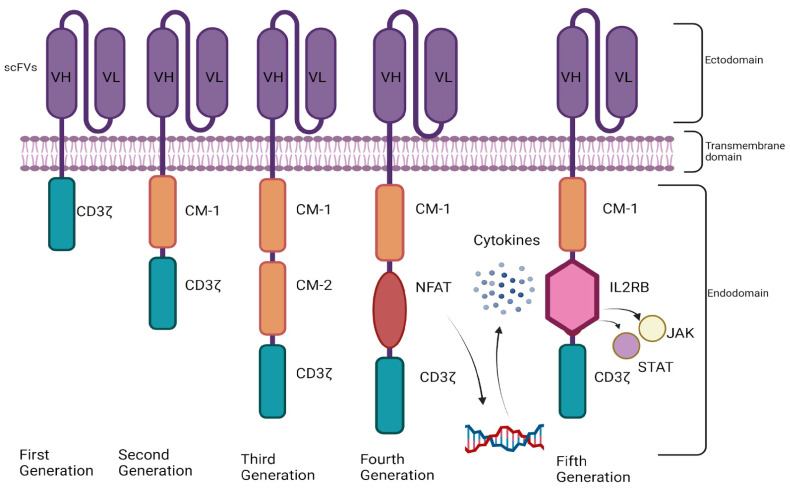
Generational advancements in chimeric antigen receptor (CAR)-T-cell constructs. CM: Costimulatory domain, NFAT: Nuclear factor of the activated T cell, JAK: Janus kinase, STATs: signal transducers and activators of transcription.

CAR engineering has progressed over the years, resulting in the development of four generations of CARs, with the fifth generation being under development [49] (Figure 2). First-generation CAR-T cells, which were first developed in 1993, contain scFvs and a single CD3 ζ chain intracellular domain; these cells are now considered obsolete owing to their limited efficacy and antitumor activity [50,51]. Second-generation CAR-T cells incorporate costimulatory domains, mainly CD28 or 4-1BB, in addition to CD3 ζ chains, which increase their T-cell activity, survival, and cytotoxicity [52,53,54,55,56]. Compared with second-generation CAR-T cells, third-generation CAR-T cells possess multiple costimulatory domains, granting them superior efficacy and persistence [57,58,59]. Fourth-generation CAR-T cells have the ability to produce or secrete cytokines due to the presence of nuclear factor of the activated T-cell (NFAT) promoters, further increasing T-cell persistence with less systemic toxicity [57,60]. Fifth-generation CAR-T cells are novel modified second-generation CAR-T cells that have improved T-cell persistence and safety profiles [49]. The endodomain of fifth-generation CARs includes a beta chain of the IL-2 receptor (IL-2 Rβ) integrated with a binding site for the transcription factor STAT3. Upon antigen recognition, triple signaling by CD3ζ, costimulatory molecules (CD-28), and cytokines (JAK–STAT3/5) occurs, resulting in T-cell activation [61].

CAR-T cells can be classified as autologous (autoCAR-T cells) or allogeneic (alloCAR-T cells) depending on their source. Compared with T cells sourced from healthy donors (alloCAR-T cells), autoCAR-T cells obtained from patients themselves can overcome immunological rejection, but other challenges may be encountered in their use, such as lengthy production timelines (typically 1–2 weeks) and reduced cytotoxicity. Conversely, challenges such as host versus graft disease (HvGD) and graft versus host disease (GvHD) are encountered when alloCAR-T cells are used [62]. However, recent clinical findings suggest that, compared with alloCAR-T-cell therapy, autoCAR-T-cell therapy has superior efficacy in the treatment of B-cell lymphomas [63,64,65,66].

**Figure 3:**
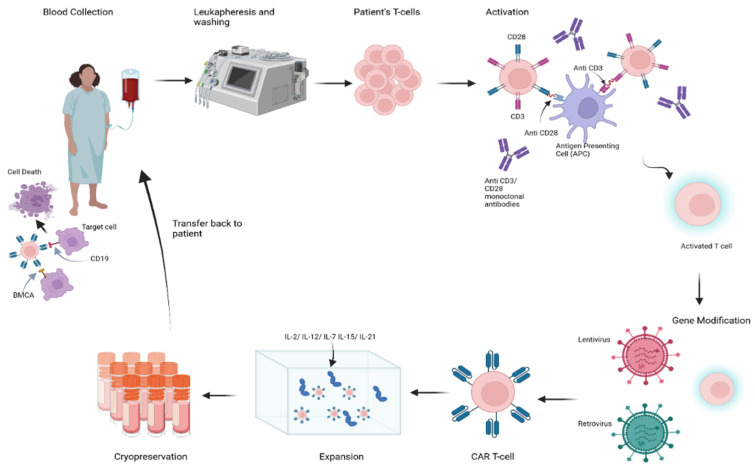
Process of chimeric antigen receptor (CAR) T-cell therapy administration for SLE patients.

CAR-T-cell production is a sequential process in which common steps are followed across different manufacturing environments [67]. This process begins with the collection of white blood cells from a patient, preceded by the cessation of all immunosuppressants except for low-dose prednisone at least 3 weeks prior [68]. The apheresis product is then washed and activated via artificial antigen-presenting cells (aAPCs) or beads coated with mAbs targeting CD3/CD28 [69]. The T cells used can be either CD4+ or CD8+ T cells; the use of CD8+ T cells is favored over the use of autoreactive CD4+ T-helper cells, although CD8+ T cells have been associated with a greater risk of CAR-T-cell exhaustion [70]. The preparation of CAR-T cells involves incubating activated T cells with a genetically modified viral vector (lentivirus or retrovirus) containing the CAR gene [71]. Upon attachment of the viral vector to the cells, the vector delivers RNA encoding the CAR. This RNA undergoes reverse transcription into DNA, which is integrated into the T-cell genome. The integrated DNA is subsequently transcribed and translated, resulting in the expression of the CAR on the cell surface [67]. Genetically modified CAR-T cells are subsequently expanded in vitro in the presence of growth factors such as IL-2, IL-12, IL-7, IL-15, and IL-21, which yields billions of cells. This volume of cells is then adjusted to an infusible volume, and the cells are cryopreserved until they are ready for administration to the patient [67,72]. Ex vivo expansion can take 9–14 days; however, a shorter culture time, in addition to fewer memory T cells in the final CAR-T-cell product, can increase the potency and efficacy of CAR-T cells [73,74]. Figure 3 shows the schematic steps of the process of CAR-T-cell therapy administration for SLE patients.

In preparation for CAR-T-cell infusion, patients undergo lymphodepletion via fludarabine at a dose of 25 mg per square meter of body surface area per day on days −5, −4, and −3 and cyclophosphamide at a dose of 1000 mg per square meter on day −3 [10]. This regimen is the most frequently used regimen by clinicians, although a specific standardization of this regimen is lacking. A total of 1 × 10^(6)^–1.1 × 10^(6)^ CD19 CAR-T cells per kilogram of body weight were then administered on day 0 [7,9,10]. After infusion, CAR-T cells can persist for years and maintain long-term remission [75].

## 4. Application of CAR-T-Cell Therapy in the Management of SLE

CAR-T-cell therapy has transformed the treatment of B-cell hematological malignancies.

The success in this field led to the approval of 6 CAR-T-cell products by the European Medicines Agency (EMA) and the US FDA in 2023 [7]. Given this success, the use of CAR-T-cell therapy in the management of lupus has attracted the interest of numerous researchers (Table 1 and Table 2).

In lupus-prone mixed New Zealand and MRL/MpJ-Fas (lpr) mice, treatment with anti-CD20 mAbs resulted in delayed disease onset, reduced T-cell activation, and slower clinical progression [83]. Nevertheless, these model animals exhibited incomplete B-cell depletion, particularly in the bone marrow and spleen. This incomplete depletion was attributed to B-cell resistance, potentially due to the production of anti-drug antibodies in addition to impaired IgG-mediated phagocytosis given the high abundance of autoreactive antibodies and immune complexes. Consequently, a higher dose and more frequent administration were required to achieve deeper B-cell depletion [84]. In contrast, CD19-targeted CAR-T-cell therapy used in the same model mice resulted in persistent depletion of CD19+ B cells in addition to clinical and serological responses [85].

Mougiakakos et al. [9] reported the first successful use of CAR-T-cell therapy for SLE. They described a 20-year-old woman with severe, refractory lupus characterized by class IIIA lupus nephritis, serositis, rash, arthritis, and a history of Libman–Sacks endocarditis. After conventional therapies (including cyclophosphamide, mycophenolate mofetil, and tacrolimus) and B-cell targeted therapies (belimumab and rituximab) failed, the patient was given autoCAR-T-cell therapy. Within 5 weeks postinfusion, clinical and serological remission were achieved, as evidenced by double-stranded DNA (dsDNA) seroconversion (from over 5000 U/mL to 4 U/mL), resolution of proteinuria (from 2000 mg/g creatinine to less than 250 mg/g), and normalization of C3 and C4 levels. No neurotoxicity, cytokine release syndrome (CRS), or prolonged cytopenia was reported. Sustained B-cell depletion followed initial CAR-T-cell expansion postinfusion. Similar results were reported by Taubmann et al. [12] when they used CD19-targeted CAR-T-cell therapy for a 32-year-old female with severe refractory lupus who achieved low disease activity at 3 months.

Furthermore, Mackensen et al. [8] conducted a larger-scale application of CAR-T-cell therapy in five patients with severe refractory SLE. Mackensen et al. [8] used a protocol similar to that of Mougiakakos et al. [9], although with a slightly lower volume of CAR-T cells infused, at 1 × 10^6^ cells per kg, than the 1.1 × 10^6^ cells per kg volume infused by Mougiakakos et al. [9]. After infusion, CAR-T cells expanded rapidly, accounting for 11% to 59% of all circulating T cells by day 9. Rapid B-cell depletion begins on day 2 and persists [8]. Drug-free remission (according to the Definition of Remission in Systemic Lupus Erythematosus (DORIS) criteria) was achieved in five patients at three months. One patient experienced a resolution of cardiac valve fibrosis and lung involvement. The B-cell population was reconstituted within a median of 110 days, with no cases of relapse. While the reappearance of B cells was associated with relapse in leukemic patients treated with CAR-T-cell therapy, the reappearance of B cells was not associated with relapse in SLE patients, who remained in remission during this limited follow-up period [86]. The re-emerged B cells had a different immunophenotype than those before CAR-T-cell infusion, being primarily CD21 + CD27– naïve cells, with low numbers (or absent) of CD21 + CD27+ memory B cells, CD38 + CD20− plasmablasts, and CD11c + CD21lo activated memory B cells, which are typically expanded in SLE. This finding indicated a profound reset of the immune system despite the reappearance of B cells and the targeting of tissue-resident B cells. Grade 1 CRS was observed in three patients, but only one required tocilizumab. Moreover, the lifespan of CAR-T cells was much lower than that reported in the hematology literature [75].

Another series of seven patients with severe refractory lupus were treated with CAR-T-cell therapy by Taubmann et al. [11] in Germany. This group of patients consisted of six females and one male aged between 19 and 39 years. All patients had multiorgan disease, including renal disease, and a median of seven prior treatments had failed. The number of CAR-T cells expanded, peaking on day 9, similar to what was observed by Mougiakakos et al. [9] and Mackensen et al. [8]. This expansion coincided with B-cell depletion, which lasted for a median of 120 days. Drug-free remission (per DORIS criterion) was achieved in all patients and lasted at least 22 months, despite B-cell population reconstitution [11].

Autologous CD19 CAR-T-cell therapy has also demonstrated efficacy in inducing remission in 15 patients with refractory autoimmune diseases, including 8 with SLE, 3 with idiopathic inflammatory myositis (IIM), and 4 with SSc [10]. These patients had active disease despite receiving at least two immunosuppressive therapies. Patients were followed up for a median of 12 months (2–28 months) after CAR-T-cell infusion. Three months after CAR-T-cell therapy, drug-free remission was achieved in all patients, although B-cell population reconstitution was observed in 12 out of 15 individuals. An SLE Disease Activity Index (SLEDAI) of 0 was achieved in patients with lupus. All patients with IIM experienced substantial symptom improvement and creatine kinase level normalization. Three SSc patients showed decreased disease activity according to the European Alliance of Associations for Rheumatology (EULAR) Disease Activity Index (DAI) after 3 months of follow-up. All 15 patients experienced CRS, but only 6 required tocilizumab for CRS management. Additionally, one patient developed immune-effector cell-associated neurotoxicity syndrome (ICANS), which manifested as vertigo.

Despite the limited use of CAR-T-cell therapy in the management of pediatric acute lymphocytic leukemia (ALL) [87], this therapy has been used to treat a 15-year-old female with severe refractory SLE [79]. The patient had rapidly progressive disease, leading to severe nephritis and nephrotic-range proteinuria necessitating hemodialysis despite receiving multiple immunosuppressive therapies, including B-cell targeted therapy. CAR-T cells were ultimately administered due to an insufficient response to treatment. The patient demonstrated a resolution of lupus symptoms, including arthritis. CAR-T-cell therapy achieved seroconversion within 6 weeks, and the patient became hemodialysis free after 3 weeks. While proteinuria persisted at 3.4 g/day, urinalysis revealed no evidence of nephritis, and proteinuria was attributed to irreversible damage. The therapy was well tolerated, with only grade 1 CRS, and the patient resumed her academic activities 4 months after therapy.

Compared with conventional CAR-T cells, YTB323 is a novel type of CAR-T-cell therapy that involves an innovative process called T-Charge TM [88]. Compared with traditional CAR-T-cell manufacturing methods, this process reduces the ex vivo culture time to approximately 24 h and the manufacturing process to less than 2 days [73,74].

After YTB323 showed efficacy in the treatment of diffuse large B-cell lymphoma (DLBCL) [88], it was recently utilized in an open-label, single-arm, multicenter phase I/II study involving 3 participants with severe refractory SLE. Preliminary efficacy data suggest improvements in the SLEDAI, Physician’s Global Assessment (PhGA) score, proteinuria, anti-dsDNA, and complement levels [78]. No serious adverse events, including ICANS or death, were reported. However, adverse events, including cytomegalovirus (CMV) reactivation, CRS, cytopenia, and hypogammaglobulinemia, were observed at varying frequencies among the study participants.

## 5. CAR-T-Cell Therapy-Related Toxicity

CRS, or cytokine-associated cytotoxicity, is an inflammatory response that stems from the activation and proliferation of T cells, complicating CAR-T-cell therapy in 42% to 93% of patients receiving this treatment [89]. Although the occurrence of CRS reflects CAR-T-cell therapy efficacy, CRS can be associated with deleterious outcomes [68,90,91]. CRS can begin within the first 1–4 days of CAR-T-cell infusion, depending on its severity. Typically, more severe episodes tend to manifest earlier [89]. The levels of laboratory markers, such as C-reactive protein (CRP) and ferritin, are significantly elevated, in addition to elevated cytokine levels, similar to those observed in hemophagocytichistiocytosis (HLH) and macrophage activation syndrome (MAS), including IL-6, IFN-γ, IL-10, soluble interleukin-2 receptor, MCP-1, and MIP1B [92]. There is a positive correlation between the development and severity of CRS and the burden of leukemic cells [68,90]. However, the degree of cytokine elevation does not necessarily reflect the severity of CRS [92,93]. Current understanding of cytokine dynamics in CRS, recognizes monocytes and macrophages as key contributors to the release of inflammatory mediators, including IL-6, IL-1, ferritin, and IFN-γ. IL-6, in particular, plays a critical role in regulating hematopoiesis, as demonstrated in multiple studies [94,95,96,97]. Both IL-6 and IFN-γ are potent pro-inflammatory cytokines that drive macrophages to secrete TNF-α and produce reactive oxygen species, ultimately leading to organ damage in CRS [98,99]. Elevated levels of cytokines such as IFN-γ, IL-6, IL-8, IL-10, and IL-15 have been correlated with more severe CRS manifestations [90,100]. Buechner et al. observed increased ferritin levels in patients with severe (grade 3 and 4) CRS [101]. Hematologic toxicities associated with CRS have been linked to biomarkers such as TNF-α and IL-6, which are predictive of severe CRS [90]. Additionally, Zhou et al. demonstrated that the severity of cytopenia following CAR-T cell therapy correlates with peak levels of IL-6, IFN-γ, CRP, and ferritin [102]. High IL-6 levels and elevated baseline β2-microglobulinhave emerged as independent risk factors for prolonged anemia, while high baseline IL- 2 levels are associated with long-term thrombocytopenia [103]. Furthermore, cardiac toxicity following CAR-T therapy has been linked to early cytokine peaks, particularly of IL-6, ferritin, and IFN-γ, which contribute to endothelial injury [104]. Whether profiling of these or other cytokines a priori can identify patients at risk for CRS has not been well studied.

CRS can be self-limited and mild, manifesting as fever or myalgia, or it can be severe, causing respiratory compromise, coagulopathy, liver dysfunction, cytopenia, or hemodynamic instability [105]. The cardiac dysfunction observed in some patients with CRS following CAR-T-cell therapy has also been reported to resemble that observed in sepsis-associated cardiomyopathy or Takotsubo cardiomyopathy but is typically reversible [91]. Ruling out infection is crucial, as many symptoms of CRS mimic sepsis, and delayed identification can result in poor outcomes and death [106].

While anecdotal reports about the association between CAR-T-cell infusion and CRS exist, a unified definition is lacking [93,107,108]. In 2014, Davila et al. [93] proposed the CRS criteria on the basis of data obtained from a cohort of 16 patients diagnosed with relapsed or refractory B-cell acute lymphoblastic leukemia who were treated with autologous anti-CD19 CAR-T cells [105]. These criteria aim to identify patients with severe CRS who require closer observation and earlier pharmacological treatment but to avoid premature treatment in patients with milder CRS, as pharmacological treatment could affect the efficacy of CAR-T-cell therapy. Severe CRS criteria included fever (body temperature ≥ 38 °C) for at least three consecutive days, along with at least one sign of toxicity, including hypotension requiring one or more intravenous vasoactive pressors; hypoxia (partial pressure of oxygen (PO_2_) < 90%); or neurological disorders, in addition to the elevation of either two cytokines with maximum fold changes of at least 75 or one cytokine with a maximum fold change of at least 250. The levels of 39 cytokines were evaluated in the present study, but only 7 were deemed relevant to CRS: IFN-γ, IL-5, IL-6, IL-10, Flt-3 L, fractalkine, and GM-CSF [105,108,109].

The National Cancer Institute Common Terminology Criteria for Adverse Events, modified by Lee et al. [68], propose another grading system for CRS, categorizing it into five severity levels from grades 1–5 [68,110]. The Grade 1 CRS includes fever and mild constitutional symptoms. Grade 2 CRS involves hypoxemia requiring low-flow oxygen (fraction of inspired oxygen (FiO_2_) < 40%), hypotension responsive to fluids or a low dose of a single vasopressor, or grade 2 organ toxicity. Grade 3 CRS includes hypoxemia requiring high-flow oxygen (FiO_2_ ≥ 40%); hypotension requiring a high dose of a single vasopressor or multiple vasopressors; grade 4 transaminitis; or grade 3 organ toxicity, such as coagulopathy and renal or cardiac dysfunction. Grade 4 CRS consists of life-threatening symptoms, the need for ventilator support, or grade 4 organ toxicity (excluding transaminitis) [91]. Grade 5 is the most severe, indicating patient death [68]. Notably, IL-6 regulates the synthesis of plasma CRP in hepatocytes [111]. Since direct measurement of cytokine levels including IL-6 is technically challenging and time-consuming, the CRP level can serve as an indirect marker of CRS related cytokine elevation and can be used as a predictive marker of the risk for the development of severe CRS and for monitoring patient response to treatment [105].

The management of CRS consists of supportive measures, but additional therapy might be needed in severe cases. Steroids can be used in the management of CRS, although concerns exist concerning the prevention of CAR-T-cell proliferation and partial response [105,106]. However, CAR-T-cell persistence was observed after short-term treatment of CRS with steroids in patients with leukemia [93,111]. A commonly employed regimen is methylprednisolone at 2 mg/kg/day, which is gradually tapered over several days [68]. Owing to its effective penetration of the blood–brain barrier, dexamethasone may be utilized in patients experiencing neurological toxicity, although there is a lack of evidence favoring one agent over the other [91,112].

Since the IL-6 level peaks during T-cell proliferation, blocking IL-6 receptors with tocilizumab has proven effective in the management of severe CRS [91,105,113]. Tocilizumab is a recombinant humanized mAb against the IL-6 receptor that prevents the binding of IL-6 to both cell-associated and soluble IL-6 receptors. Tocilizumab has been approved by the FDA for the safe treatment of rheumatoid arthritis and juvenile idiopathic arthritis (JIA) [114]. The recommended dose of tocilizumab is 4 mg/kg in adults and 8 mg/kg in children, which is infused over 1 h [68,115]. Symptom improvement should be noticeable within a few hours after infusion. If improvement is not observed within 24 h, repeated dosing with tocilizumab or the addition of another immunosuppressive agent, such as corticosteroids, is recommended [91]. The efficacy of CAR-T cells was not found to be hindered by the use of tocilizumab in patients with leukemia [93,105]. Additional immunosuppressive agents to consider in the management of CRS include mAbs targeting TNFα (such as infliximab), soluble TNFα receptor (such as etanercept), or IL-1R-based inhibitors (such as anakinra). Targeting IFN-γ with emapalumab-lzsg may also offer a novel approach for early intervention in patients undergoing CAR-T therapy, particularly those with elevated IFN-γ levels. These options are considered due to their effectiveness in treating MAS, which has many similarities with CRS [116,117,118]. However, corticosteroids and tocilizumab remain the most frequently utilized immunosuppressive therapies in the management of CRS.

ICANS is another potential complication of CAR-T-cell therapy and tends to be self-limiting. ICANS typically follows CRS but is not associated with CRS severity. The mechanism underlying ICANS development remains unknown but could be related to T-cell activity or cytokine release [89]. Symptoms of ICANS vary widely and include delirium, seizure-like activity, confusion, word-finding difficulty, aphasia, and obtundation requiring mechanical ventilation in severe cases. Diagnostic evaluations, including brain imaging (computed tomography (CT) scans or magnetic resonance imaging (MRI)), cerebrospinal fluid (CSF) analysis, and electroencephalography (EEG), are usually unrevealing. While CAR-T cells have been detected in the CSF of some affected patients, this finding was not consistent across all patients [105]. Caution should be taken when using tocilizumab to treat CRS in patients with neurological dysfunction, as this drug may transiently worsen their symptoms [119,120]. Corticosteroids might be preferred in this subset of patients [91].

Best practice recommendations were released by the European Society for Blood and Bone Marrow Transplantation [121], the European Hematology Association [122], and the American Society of Clinical Oncology [123]. Overall, the treatment of CRS and ICANS includes supportive care with fluid replacement, oxygen supplementation, treatment of possible infections, and vasopressors if needed. Tocilizumab can be used for the treatment of severe CRS, whereas steroids can be effective for both CRS and ICANS. The management of refractory cases can include tocilizumab in combination with other immune modulators, e.g., anakinra (IL-1 receptor antagonist) and siluximab (monoclonal anti-IL-6) [124].

Hematological toxicities, such as anemia, thrombocytopenia, and leukopenia with resulting hypogammaglobulinemia, have been associated with CAR-T-cell therapy [125]. Severe complications, including disseminated fungal infection [126] and lethal cerebral hemorrhage [125], have also been reported. To mitigate the risk of infection associated with severe hypogammaglobulinemia, intravenous immunoglobulins can be used [66].

Cellular toxicities can be categorized into “on-target-on-tumor”, “on-target-off-tumor” and “off-target” toxicities [127]. These types of toxicity are more commonly observed in the management of hematological malignancies via CAR-T-cell therapy. “On-target-on-tumor” toxicity is the most common type of toxicity observed after CAR-T-cell administration. This type of toxicity results from cytokine release and tumor cell necrosis mediated by effector CAR-T-cell activation, which causes CRS and tumor lysis syndrome (TLS) [125]. TLSs are characterized by metabolic derangements and electrolyte imbalances due to the rapid destruction of tumor cells [86,128]. Early detection and management are essential for improved patient outcomes [129]. “On-target-off-tumor” toxicity results from CAR-T cells recognizing target antigens on normal cells, leading to their destruction [130,131]. The identification of antigens strictly present on tumor cells is needed to overcome this challenge, but this can be difficult to achieve. “Off-target” toxicity occurs when CAR-T cells target unintended epitopes or activate them independently of their specificity [132]. Off-target toxicity has not been observed in CAR-T-cell trials thus far. However, two cases of cardiotoxicity were reported with the use of high-affinity T-cell receptors against melanoma antigen family A, 3 (MAGE-A3) in the treatment of melanoma and myeloma. The unexpected cross-reactivity with the muscle protein titin resulted in lethal cardiomyopathy and cardiogenic shock due to the high potency of these T cells [133,134].

Immunogenicity is another complication that can be observed with CAR-T-cell therapy. Most of the CAR antigen recognition region is retrieved from murine antibodies [135]. Infusion of these CAR-T cells can trigger an IgE-mediated allergic response, resulting in anaphylaxis [136]. For example, anti-mouse antibodies were detected in a patient with mesothelioma who developed cardiorespiratory compromise following the 3rd dose of mesothelin-specific CAR-T cells [137].

Oncogenesis caused by the genomic integration of a viral vector into the human genome is another long-term concern with CAR-T-cell therapy. The FDA has recommended extended follow-up of patients receiving CAR-T cells engineered with integrated vectors to monitor these patients for adverse events, including cancer [138]. In November 2023, the FDA reported 22 cases of second primary malignancies, including T-cell lymphoma, T-cell large granular lymphocytosis, peripheral T-cell lymphoma, and cutaneous T-cell lymphoma, out of 8000 total records in the FDA Adverse Event Reporting System (FAERS) database as of December 2023 [138,139]. These cases were associated with five out of the six CAR-T-cell products. In 14 of these patients, the cancer manifested within 2 years after CAR-T-cell therapy. The CAR transgene was detected in the malignant clone in three of these cases, indicating a potential association between T-cell malignancy and the CAR-T-cell product [140]. In January 2024, the director of the FDA Center for Biologics Evaluation and Research (CBER) suggested that the risk–benefit profile of CAR-T cells is not in question in oncology and that research and development programs for CAR-T-cell therapies in autoimmune diseases should move forward [141]. In April 2024, the FDA required the addition of a box warning for T-cell malignancy to approved CAR-T-cell products (CD19- and BCMA-targeted) when used in patients treated for hematological malignancies [142]. Notably, many unknown risk factors, including age and immune status, can contribute to the development of secondary malignancies. The current recommendation is lifelong monitoring for secondary malignancies in patients who have received these therapies [143]. However, one published case of confirmed CAR+ T-cell malignancy may have been due to genetic mutations present in the patient before their CAR-T cells were manufactured [144]. As of April 2024, the EMA has investigated 27 cases of T-cell lymphoma or leukemia [139,145]. The EMA notes the treatment of over 40,000 patients with CAR-T-cell therapies worldwide. Furthermore, no cases of malignancy have been reported in autoimmune diseases such as SLE to date. Hematology and oncology literature suggests that secondary malignancies associated with CAR-T cell therapy remain poorly understood, and the overall risk is relatively low, although this continues to be an area of active research [146]. The FDA currently mandates a 15-year follow-up for all patients with autoimmune diseases enrolled in CAR-T cell trials [147,148]. These guidelines, adapted from CAR-T cell therapy protocols for cancer, recommend a follow-up schedule starting with monthly evaluations, transitioning to every three months, and eventually annual assessments until the 15-year mark. This extended monitoring is critical for detecting any long-term adverse effects, including potential malignancies related to the therapy.

Another safety consideration regarding CAR-T-cell therapy is its effect on the immune response to vaccination. A reduction in antibody titers against measles, mumps, rubella, varicella-zoster virus, tetanus, diphtheria, and pneumococci was observed in several studies [149,150,151,152], but most of these patients maintained seroprotection against the aforementioned infections. Mackensen et al. [8] also noted no significant decline in antibody titers against these infections following CAR-T-cell therapy compared with before therapy, which indicates that CAR-T cells spare CD19-negative plasma cells [153]. Although the optimal timing of vaccination relative to CAR-T-cell therapy is still uncertain, Walti et al. [154] reported that 40% of patients with B-cell malignancies elicited responses to at least one quadrivalent influenza vaccine strain before CAR-T-cell therapy, whereas 31% elicited responses afterward.

## 6. Conclusions and Future Directions

The complexity of SLE pathogenesis poses challenges for the development of curative therapies. B cells are critical in lupus pathogenesis, with autoantibody production starting many years before clinical symptoms appear. CAR-T cells, as discussed in this review, affect multiple aspects of the immune response, with deep and sustained eradication of target antigen-expressing B cells and resetting of the immune system demonstrated. After receiving cd19-CAR-T-cell therapy, patients exhibit a completely naïve B-cell pattern, with very few memory cells. Furthermore, this therapy depletes plasmablasts, and activated memory B cells, which are associated with lupus activity and flares. Interestingly, B-cell receptor sequencing and heavy chain analysis revealed a predominantly nonclass-switched population of B cells, which are IgM- and IgD-positive and lack heavy chains for IgG and IgA. It is anticipated these will likely re-emerge later upon exposure to infections and vaccinations.

This approach targets various B cell related pathogenic pathways, keeping patients free from immunosuppression and in remission, thus underscoring the heterogeneity of the disease. CAR-T-cell therapy has helped reframe the traditional approach from merely suppressing the disease to potentially curing it. Nevertheless, more information is needed regarding the efficacy and safety of CAR-T-cell therapy, and many questions remain unanswered about its use in SLE patients (Table 3). As CAR-T therapy evolves for treating autoimmune diseases, effective toxicity monitoring will be essential for optimizing both the safety and efficacy of treatment. The identification and measurement of key biomarkers cytokines along with comprehensive monitoring of clinical parameters, may enable the early detection of toxicity, enhance severity assessment, and improve clinical management. This biomarker-driven approach offers the potential for more personalized treatment strategies, as opposed to relying on single-marker assessments. A multidisciplinary team, including hematology and rheumatology specialists, is essential for CAR-T-cell optimization, patient risk stratification, and improving CAR-T-cell therapy administration. Currently, numerous clinical trials are underway, with some actively enrolling patients with SLE and other autoimmune diseases, paving the way for the utilization of CAR-T-cell therapy in the field of rheumatology (Table 4).

## Figures and Tables

**Table 1:** Case reports and case series of chimeric antigen receptor (CAR)-T-cell therapy for systemic lupus erythematosus (SLE).

Author	Year	Number of Patients	Age (Years)	Sex	Disease Activity Score	Organ/Tissue Involved	Previous Treatment	CAR-T-Cell Protocol	Response to CAR-T-Cell Therapy	Complications
Mougiakakos et al. [9]	2021	1	20	F	SLEDAI-2K score: 16	Kidney, serous tissues, skin, joints, heart	GCs, HCQ, cyclophosphamide, MMF, tacrolimus, belimumab, RTX	Day −5 to day −3: fludarabine 25 mg/m^2^/d Day −3: cyclophosphamide 1000 mg/m^2^/d Day 0: 1.1 × 10^6^ CD19-directed CAR-T cells/kg	-SLEDAI-2K score of 0 after 6 weeks -Proteinuria normalization (<250 mg protein/g creatinine) after 1 month -Complement level normalization in 1 month -Anti-dsDNA seroconversion in 1 month	None
Mackensen et al. [8]	2022	5	18–22	F: 4 M: 1	SLEDAI-2K score: 8–16	Skin, joints, kidneys, heart, serous tissues, muscle, bone marrow, lungs	GCs, HCQ, MMF, AZA, RTX, cyclophosphamide, tacrolimus, belimumab, MTX, leflunomide	Day −5 to day −3: fludarabine 25 mg/m^2^/d Day −3: cyclophosphamide 1000 mg/m^2^/d Day 0: 1.0 × 10^6^ CD19-directed CAR-T cells/kg	-SLEDAI-2K score of 0 in 4/5 patients after 3 months -Proteinuria normalization (<300 mg protein/g creatinine) after 3 months -Complement level normalization -Anti-dsDNA seroconversion	CRS grade 1 in 3/5 patients
Müller et al. [10,76]	2023 2024	15 SLE 8/15	18–38	F: 7 M: 1	SLEDAI-2K score: 9.3 to 16	Kidney, skin, joints, bone marrow, lungs, heart	GCs, HCQ, MMF, AZA, RTX, cyclophosphamide, tacrolimus, belimumab, MTX, leflunomide, bortezomib, upadacitinib, ustekinumab, lenalidomide, thalidomide, interleukin-2	Day −5 to day −3: fludarabine 25 mg/m^2^/d Day −3: cyclophosphamide 1000 mg/m^2^/d Day 0: 1.0 × 10^6^ CD19-directed CAR-T cells/kg	-SLEDAI-2K score of 0 in 8/8 patients after 3 months -Proteinuria resolution after 3 months -Complement level normalization -Anti-dsDNA seroconversion	CRS grade 1 in 5/8 patients Hypogammaglobulinemia in 3/8 patients Pneumonia (requiring hospitalization) 1/8 patients
Wang et al. [77]	2024	13	16–58	F: 10 M: 3	SLEDAI-2K score: 4° 16	Kidneys, bone marrow, skin, joints, heart. Patient 1 and Patient 2 had DLBCL	GCs, HCQ, MMF, cyclophosphamide, belimumab, tacrolimus, thalidomide	Patient 1 and Patient 2: cyclophosphamide (0.3 g per m^2^) and fludarabine (0.03 g per m^2^) Patient 3-Patient 13: cyclophosphamide (0.3 g per m^2^) Day 0: 3 × 10^6^ BCMA/CD19-directed CAR-T cells/kg except Patient 11 received a dose of 1.5 × 10^6^ cells/kg	-Complete remission of DLBCL was achieved in Patient 1 and Patient 2 -SLEDAI-2K score of 0 in 11/13 patients -Complement level normalization -Anti-dsDNA seroconversion in 12/13 patients within 3 weeks Proteinuria normalization (<250 mg protein/g creatinine) after 6 months in 8/11 patients with lupus nephritis	CRS grade 1 in 9/13 patients Hypogammaglobulinemia in 10/13 patients
Hernández et al. [78]	2024	3	38–50	F 2 M 1	SLEDAI-2K score: 12 to 22	Kidneys, joints, skin, pleura, vasculature	N/A	Day −14: lymphodepletion with cyclophosphamide and fludarabine. Day 1: 12.5 × 10^6^ CD19-directed CAR-T cells	-SLEDAI-2K score reduction by 50% at 2 months -Improvement in (PhGA) score, anti-dsDNA levels, proteinuria and complement levels	CRS grade 1 or 2 in 2/3 patients Hypogammaglobulinemia in 2/3 patients CMV reactivation in 1/3 patients
Taubmann et al. [12]	2024	1	32	F	SLEDAI-2K score: 10	Pericardium (effusion), kidneys, bone marrow, brain, skin	GCs, HCQ, MMF, tacrolimus, belimumab, cyclophosphamide, rituximab	Day −5 to day −3: fludarabine 12.5 mg/m^2^/d Day −3: cyclophosphamide 500 mg/m^2^/d. Day 0: CAR-T-cell volume not specified	-SLEDAI-2K score of 0 after 25 days -Proteinuria normalization (<300 mg protein/g creatinine) -Anti-dsDNA seroconversion	None
Krickau et al. [79]	2024	1	15	F	SLEDAI-2K score: 23	Skin, joints, kidneys	GCs, HCQ, azathioprine, MMF, belimumab, cyclophosphamide, plasma exchange	Day −5 to day −3: fludarabine 12.5 mg/m^2^/d Day −3: cyclophosphamide 500 mg/m^2^/d. Day 0: 1.0 × 10^6^ CAR-T cells/kg	-SLEDAI-2K score of 0 in 6 months -Hemodialysis free after 3 weeks -Complement level normalization in 6 weeks -Anti-dsDNA seroconversion in 6 weeks -Proteinuria decreased from 10,717 mg/g/day to 3400 mg/kg/day after 6 months	CRS grade 1
Podoll et al. [80]	2024	2	18, 28	F	SLEDAI-2K score: 12–19	Kidneys (class IV lupus nephritis), bone marrow	N/A	Day –7 to –5: -fludarabine (30 mg/m^2^/ day) -cyclophosphamide (300 mg/m^2^/day) Day 0: Patient-1: 0.5 × 10^8^ Patient-2: 1.0 × 10^8^ CD19-directed CAR-T cells	-SLEDAI-2K score reduction Patient 1: from 19 to 8 on Day 90 Patient 2: from 12 to 10 on Day 28 -Proteinuria improvement Patient 1: from 1.4 g/g/day to 0.5 g/g/day on Day 90 Patient 2: from 1.3 g/g/day to 0.6 g/g/day on Day 28 -Complement level elevation -Anti-dsDNA level reduction	CRS grade 1 in 2/2 patients
Marasco et al. [81]	2024	1	15	F	SLEDAI-2K score: 22	Kidneys, serous tissues, skin, bone marrow, lungs (PAH).	GCs, HCQ, MMF, RTX, cyclophosphamide.	lymphodepletion: cyclophosphamide (1500 mg/m^2^). fludarabine (90 mg/m^2^), −1 × 10⁶ CD19-directed CAR-T cells/kg	-SLEDAI-2K Score: 2 at week 6 -Urinalysis: Normal results at week 3. -Complement levels: normalized at week 6. -ANA and anti-dsDNA titers: significantly decreased at week 8. -Right ventricular systolic pressure and NT-ProBNP levels: Normal at week 2.	-CRS grade 1 -Cytopenia (transient)

ANA: antinuclear antibody; AZA: azathioprine; CMV: cytomegalovirus; CRS: cytokine release syndrome; DLBCL: diffuse large B-cell lymphoma; dsDNA: double-stranded DNA; F: female; GCs: glucocorticoids; HCQ: hydroxychloroquine; M: male; MMF: mycophenolate mofetil; MTX: methotrexate; N/A: not applicable; PAH: pulmonary artery hypertension; PhGA: Physician Global Assessment; RTX: rituximab; SLEDAI-2K: Systemic Lupus Erythematosus Disease Activity Index 2000.

**Table 2:** Abstract on chimeric antigen receptor (CAR)-T-cell therapy for systemic lupus erythematosus (SLE) presented at EULAR 2024 [82].

Abstract #	Title, Author, and Location						
POS0054	An open-label, single-arm, multicenter study to evaluate elmacabtagene autoleucel, the CD-19 directed CAR-T-cell therapy, for active systemic lupus erythematosus in china. Hu et al., China						
# of patients	Disease/activity	Age	Sex	CAR-T-cell protocol/method	Previous medications	Response (follow-up duration)	Complications
3	SLE Organs involved: skin, kidneys, bone marrow, joints	21–36 years	F	25 × 10^6^ CAR T cells after lymphodepleting therapy (Cyclophosphamide and Fludarabine)	GCs, HCQ, MMF, tacrolimus, MTX, telitacicept, belimumab.	Clinical and Serological Responses: -SELENA-SLEDAI decreased to 0–1 -SRI-4 achieved in all patients -LLDAS achieved in two patients -Proteinuria improved -Autoantibodies decreased -C3 levels elevated Cellular Response: -Cellular expansion with median peak concentration (C_max) of 19.72 cells/μL between 8–22 days postinfusion. -Complete B-cell depletion was observed reaching the nadir between Day 8–11.	-CRS G1 in 1 patient and G3 in 1 patient -Cytopenia (1 patient) -Infection, MAS and effusion (1 patient)
POS0340	Effects of CAR-T-cell treatment on b-cell immunity in systemic autoimmune diseases. Bucci et al., Germany						
12	8 SLE 2 IIM 2 SSc	N/A	N/A	CD19 CAR T-cell therapy	N/A	Clinical and Serological Responses: N/A Cellular response: -Reconstituted B cells had a naïve phenotype, with reduced CD19 + CD27+ memory B cells. -Minimal increase in memory B cells, mostly preswitched IgD+ CD27+. -Plasmablasts and activated CD11c+ memory B cells disappeared in SLE patients. -Increase in immature CD38+ B cells at 4 months, declining later. -Single-cell sequencing showed reduced expression of class-switched heavy chains and disease-associated chains, with increased IGHM and IGHD expression.	N/A
OP0027	Long-term safety and efficacy of CAR-T-cell treatment in severe and refractory autoimmune disease cases. Taubmann et al., Germany						
15	8 SLE 4 SSc 3 IIM	18–60 years	F: 10 M: 5	1.0 × 10^6^ CD-19 CAR T cells after lymphodepleting therapy (Cyclophosphamide and Fludarabine)	N/A	Clinical and Serological Responses: SLE: DORIS: remission was achieved in all SLE patients IIM: ACR/EULAR: a major response was achieved in all patients SSc EUSTAR activity index: decreased in all patients. -Drug-free remission achieved in all patients Cellular Response: -N/A	-CRS (G1: 8 patients, G2: 1 patient) -ICANS (grade 1): 1 patient) -Late-stage neutropenia in 1 patient. -Infections (Pneumonia/upper respiratory tract infections.)
POS0046	Preliminary results of an open-label, multicentre, phase 1/2 study to assess the safety, efficacy and cellular kinetics of ytb323 (rapcabtagene autoleucel), a rapidly manufactured CAR-T-cell therapy targeting CD19 on b cells, for severe refractory systemic lupus erythematosus. Cortés-Hernández, et al., Spain						
6	SLE	N/A	N/A	YTB323 12.5 × 10^6^ CD-19 CAR T cells after lymphodepleting therapy (Cyclophosphamide and Fludarabine)	N/A	Preliminary efficacy data for the first 3 patients showed: Clinical and Serological Responses: -Significant reductions in SLE Disease Activity Index (SLEDAI) and Physician’s Global Assessment (PhGA). -Improvements in disease biomarkers such as autoantibodies, complement levels, and proteinuria. Cellular response: -Peak CAR T-cell expansion 13–21 days postinfusion. -Deep B-cell depletion followed by B-cell recovery.	-CRS (G1 or G2 in 4 patient) -Cytopenia (G3 and G4) in all patients -Hypogammaglobulinemia. -Infection (pneumonia in 1 patient)
POS0030	Safety and preliminary efficacy of CD19 CAR-T-cell treatment in rheumatic disease: data from the first part of the phase i/ii castle basket study (CASTLE study) Schett et al., Germany						
8 (1st part) 16 (2nd part)	5 SLE 3 SSc 1 IIM	20–81 years	F: 6 M: 2	1.0 × 10^6^ CD-19 CAR T cells/kg body weight after lymphodepleting therapy (Cyclophosphamide and Fludarabine)	N/A	Clinical and Serological Responses: -SLE: DORIS remission achieved in three patients -IIM: ACR moderate/major response achieved in one patient -SSc: lung function maintained in 1 patient. -Drug-free remission achieved in all patients Cellular response: -Complete B-cell depletion in all patients within 10 days. -CAR-T cells expanded in all patients.	-CRS (G1: 4 patients, G2: 1 patient. -Late-stage neutropenia: 2 patients. -Infections (pneumonia, SARS-CoV-2 and CMV) that resolved upon treatment: 2 patients.
POS0464	Serum proteomic analysis identifies markers associated with anti-CD19 CAR-T therapeutic response in autoimmune diseases J. Chou et al., Germany						
8	3 SLE 3 diffuse SSc 2 DM Control: 10 HC 7 SLE 7 SSc	N/A	N/A	CD19-CAR T-cell therapy	N/A	Clinical and Serological Responses: N/A Cellular Response: -IgM, IgA, IgE: Significantly reduced at 3 months post-CD19-CAR T-cell infusion. -IgG: No significant change observed. -SLE Baseline: Elevated IFN signaling molecules (CXCL10, MX1). -SSc Baseline: Elevated markers of endothelial dysfunction (VEGF, ANG2). Downregulated Pathways Post-Therapy: HSF-1–mediated heat shock response (HSPA1A, DNAJA4), type I IFN signaling (IFIT3, ISG15). -Reduction in autoantigen PUF60, which is related to neutrophil degranulation and IL12 signaling.	N/A
POS1325	Anti-CD19 CAR-T-cell therapy for refractory childhood-onset systemic lupus erythematosus Bracaglia et al., Italy						
2	Childhood-Onset SLE Organs involved: Kidneys, lungs, heart, CNS	15 and 17 years	F	1 × 10^6^ cells/kg body weight CD-19 CAR T cells	Patient 1: GC, MMF, RTX, CYC. Patient 2: GC, MMF, CYC pulses, plasmapheresis	Patient 1: Clinical and Serological Responses: -Pulmonary hypertension improved. -C3 and C4 normalized by week 6, -Proteinuria normalized by week 4. -Renal biopsy at month 6 showed no glomerular deposits. -SLEDAI-2K normalized at month 3 with sustained drug-free remission at month 6. Cellular response: -Peak CAR-T-Cell expansion on day 12 (52.4 cells/μL). -Complete B-cell Depletion by day 7. -B-cell recovery occurred at 4 months without SLE flare. Patient 2: Clinical and Serological Responses: -Normal C3 and C4. -Markedly decreasing proteinuria -Off immunosuppression. Cellular response: -N/A	Patient 1: -CRS (G1) -Transient anemia (G2) -Transient neutropenia (G3) Patient 2: -N/A

ANA: antinuclear antibody; CAR: chimeric antigen receptor; cSLE: childhood-onset systemic lupus erythematosus; CNS: central nervous system; CRS: cytokine release syndrome; CYC: cyclophosphamide; DM: dermatomyositis; dsDNA: double-stranded DNA; F: female; G: grade; GCs: glucocorticoid; HC: healthy control; HCQ, hydroxychloroquine; ICANS: immune effector cell-associated neurotoxicity syndrome; ICU: intensive care unit; IIM-ACR: idiopathic inflammatory myopathy–American College of Rheumatology; ILD: interstitial lung disease; IVIG: intravenous immunoglobulin; LLDAS: lupus low disease activity state; LN: lupus nephritis; M, male; MAS: macrophage activation syndrome; MMF: mycophenolate mixture; MTX, methotrexate; PE: plasma exchange; PH: pulmonary hypertension; PhGA: Physician Global Assessment; RTX: rituximab; SLE-DORIS: systemic lupus erythematosus—definition of remission in SLE; SLEDAI-2k: systemic lupus erythematosus Erythematosus Disease Activity Index 2000.

**Table 3:** Research questions for future studies.

-What criteria, such as severe organ damage, life-threatening complications, or immune profiling, should be used to determine eligibility for CAR-T-cell therapy in SLE patients? -When should CAR-T-cell therapy be prescribed for SLE patients, particularly for those patients with early disease and poor predicted outcomes or patients with refractory disease or both? -How do different CAR-T-cell constructs targeting CD19 vs. BCMA or both compare in terms of efficacy and safety for treating SLE patients? -What are relative advantages/disadvantages of alternative cell-based B cell depleting strategies such as cd19CAR-NK or bispecific (CD3 × CD19) monoclonal reagents vs cd19CAR-T? -How important is seroconversion, specifically the absolute resolution of all autoantibodies? -What is the risk–benefit ratio regarding the persistence of CAR-T cells and the duration of B-cell depletion? -What are the optimal management strategies for SLE patients receiving CAR-T-cell therapy, including the use of hydroxychloroquine, immunosuppressive agents, and biologics? -In cases of relapse after CAR-T-cell therapy, which treatments should be used? -What type of concomitant immunosuppression is appropriate given the B-cell aplasia induced by CAR-T-cell therapy? -What are the optimal requirements and methods for achieving lymphodepletion, including the extent and intensity of the chemotherapy regimen? -What risk factors, including infections and malignancies, should be assessed before CAR-T-cell therapy is initiated in SLE patients? -How can the risk of malignancy be mitigated when CAR-T-cell therapy is the best option for SLE treatment? -How should the efficacy of vaccination be evaluated, how should vaccination be scheduled for SLE patients receiving CAR-T-cell therapy, and what vaccines are needed before and after treatment? -How should antimicrobial prophylaxis be managed in SLE patients with a history of severe infections receiving CAR-T-cell therapy? -What are the safety profiles of specific CAR-T-cell therapies for high-risk SLE patients? -What neurological side effects could arise from CAR-T-cell therapy in SLE patients, and how can these side effects be monitored and managed?

CAR: chimeric antigen receptor, SLE: systemic lupus erythematosus.

**Table 4:** Chimeric antigen receptor (CAR)-T-cell therapy clinical trials for systemic lupus erythematosus (SLE).

Clinical Trial	Number of Participants	CAR-T-Cell Therapy Target	Title	Study Phase	Location	Status
NCT06106906	15	CD19	A Clinical Study of CD19 CAR-T in Refractory/Moderate-to-Severe Systemic Lupus Erythematosus	Phase I Phase II	China	Not yet recruiting
NCT06340750	18	BAFF-ligand	BAFF CAR-T Cells (LMY-920) for Systemic Lupus Erythematosus	Phase I	N/A	Not yet recruiting
NCT0610689	15	CD19	A Clinical Study of CD19 Universal CAR-γδT Cells in Active Systemic Lupus Erythematosus	Phase I Phase II	China	Recruiting
NCT06150651	6	CD19	Safety of PiggyBac Transposon CAR-T cells Targeting CD-19 in Refractory Lupus.	Phase I	Thailand	Recruiting
NCT06428188	60	BCMA CD19	Sequential CAR-T Cells Targeting BCMA/CD19 in Patients with Relapsed/Refractory Autoimmune Diseases (BAH247)	Phase I Phase II	China	Recruiting
NCT06340490	24	CD19	A Study of RJMty19 in Refractory Systemic Lupus Erythematosus (SLE)	Phase I	China	Not yet recruiting
NCT05030779	9	CD19 BCMA	A Study of CD19/BCMA Chimeric Antigen Receptor T Cells Therapy for Patients with Refractory Systemic Lupus Erythematosus	Early phase I	China	Unknown
NCT05988216	12	CD19	Universal CAR-T Cells (BRL-301) in Refractory Systemic Lupus Erythematosus	N/A	China	Recruiting
NCT03030976	5	CD19	A Study of CD19 Redirected Autologous T Cells for CD19 Positive Systemic Lupus Erythematosus (SLE)	Phase I	China	Unknown
NCT06350110	75	CD19 BCMA	Fourth-gen CAR-T Cells Targeting BCMA/CD19 for Refractory Systemic Lupus Erythematosus (SLE) (BAH242)	Phase I Phase II	China	Not yet recruiting
NCT06347718	24	CD19	CAR-T Cells in Systemic B-Cell Mediated Autoimmune Disease (CASTLE)	Phase I Phase II	Germany	Recruiting
NCT06189157	29	CD19	MB-CART19.1 in Refractory SLE	Phase I Phase II	Germany	Not yet recruiting
NCT05858684	18	CD19 BCMA	Dual Target CAR-T-Cell Treatment for Refractory Systemic Lupus Erythematosus (SLE) Patients	Early phase I	China	Recruiting
NCT06153095	30	CD19 CD20	A Study of IMPT-514 in Active Refractory Systemic Lupus Erythematosus (SLE)	Phase I Phase II	United States	Recruiting
NCT06342960	32	CD19	A Study of Anti-CD19 Chimeric Antigen Receptor T-Cell (CD19 CAR-T) Therapy in Subjects with Refractory Lupus Nephritis (KYSA-3)	Phase I Phase II	Germany	Recruiting
NCT06429800	26	CD19	A Study to Evaluate the Safety and Preliminary Efficacy of ATA3219 in Participants with Lupus Nephritis	Phase I	Unknown	Not yet recruiting
NCT05474885	15	CD19 BCMA	BCMA-CD19 cCAR-T-Cell Treatment of Relapsed/Refractory Systemic Lupus Erythematosus (SLE)	Phase I	China	Recruiting
NCT05938725	32	CD19	A Study of Anti-CD19 Chimeric Antigen Receptor T-Cell (CD19 CAR-T) Therapy, in Subjects with Refractory Lupus Nephritis	Phase I Phase II	United States	Recruiting
NCT0627742	24	BCMA	Refractory ANCA Associated Vasculitis and Lupus Nephritis Treated With BCMA-targeting CAR-T Cells	N/A	China	Recruiting
NCT06373081	6	CD19 CD3E	Anti-CD19-CD3E-CAR-T Cells in Relapsed/Refractory Autoimmune Disease	N/A	China	Recruiting
NCT06316791	24	CD19	Exploratory Clinical Study of CNCT19 Anti CD19 Cell Therapy in the Treatment of Refractory Autoimmune Diseases	Early phase I	China	Recruiting
NCT06222853	19	CD19	Study of Therapeutic Efficacy of Anti-CD19 CAR-T Cells in Children with Refractory Systemic Lupus Erythematosus	Phase I	China	Recruiting
NCT05765006	24	CD19	CD19-CART(Relma-cel) for Moderate to Severe Active Systemic Lupus Erythematosus	Phase I	China	Recruiting
NCT05085418	9	CD19 BCMA	A Study of CD19/BCMA Chimeric Antigen Receptor T Cells Therapy for Patients with Refractory Immune Nephritis	Early phase I	China	Recruiting
NCT05846347	15	CD19 BCMA	Phase I Clinical Study of GC012F Injection in Treatment of Refractory Systemic Lupus Erythematosus	Phase I	China	Recruiting
NCT05859997	15	CD19	Universal CAR-T Cells (BRL-301) in Relapse or Refractory Autoimmune Diseases	N/A	China	Recruiting
NCT06420154	9	CD19	The Safety and Efficacy of Anti-CD19 CAR-T Cells in Patients with Relapsed/Refractory Autoimmune Diseases	Early phase I	China	Not yet recruiting
NCT06297408	24	CD19	Relma-cel for Moderate to Severe Active Systemic Lupus Erythematosus	Phase I	Unknown	Not yet recruiting
NCT06038474	30	BCMA	Descartes-08 for Patients with Systemic Lupus Erythematosus (SLE-001)	Phase II	United States	Recruiting
NCT06294236	36	CD19	Study Evaluating SC291 in Subjects with Severe r/r B-cell Mediated Autoimmune Diseases (GLEAM)	Phase I	United States	Recruiting
NCT06462144	36	CD19 CD20	IMPT-514 in Systemic Lupus Erythematosus, Anca-associated Vasculitis, and Idiopathic Inflammatory Myopathy	Early phase I	China	Not yet recruiting
NCT06333483	12	CD19	A Study of CD19 Targeted CAR-T-Cell Therapy in Patients with Severe, Refractory Systemic Lupus Erythematosus (SLE) (CARLYSE)	Phase I	United Kingdom	Recruiting
NCT06249438	30	BCMA CD20	A Study of C-CAR168 in the Treatment of Autoimmune Diseases Refractory to Standard Therapy (CAR-AID)	Phase I	China	Recruiting
NCT05930314	12	CD19	CNCT19 Cell Injection for Refractory Systemic Lupus Erythematosus	Early phase I	China	Enrolling by invitation
NCT06465147	12	CD19	REACT-01: Reversing Autoimmunity Through Cell Therapy	Phase I	United States	Not yet recruiting
NCT05798117	24	CD19	An Open-label, Study to Assess Safety, Efficacy and Cellular Kinetics of YTB323 in Severe, Refractory Systemic Lupus Erythematosus	Phase I Phase II	United States	Recruiting
NCT06121297	12	CD19	RESET-SLE: A Phase 1/2 Open-Label Study to Evaluate the Safety and Efficacy of CABA-201 in Subjects with Active Systemic Lupus Erythematosus	Phase I Phase II	United States	Recruiting
NCT06310811	12	CD19	Anti-CD19 CAR-T-Cell Therapy in Participants with Moderate to Severe Active Systemic Lupus Erythematosus	N/A	China	Recruiting
NCT06375993	40	CD20	A Phase 1 Study of ADI-001 in Lupus Nephritis	Phase I	Unknown	Not yet recruiting
NCT05869955	129	CD19	A Study of CC-97540, CD-19-Targeted Nex-T CAR-T Cells, in Participants with Severe, Refractory Autoimmune Diseases	Phase I	United States	Recruiting
NCT06417398	10	CD19	Preliminary Clinical Study of UTAA09 Injection in the Treatment of Relapsed/Refractory Autoimmune Diseases	Early phase I	Unknown	Not yet recruiting
NCT06361745	10	CD19	Early Clinical Study of UTAA09 Injection in the Treatment of Relapsed/Refractory Autoimmune Diseases	N/A	China	Recruiting
NCT06285279	24	BCMA CD19	FKC288 in Participants with Autoimmune Kidney Diseases	Phase I	China	Recruiting

N/A: not applicable.

## Data Availability

Not applicable.

## References

[1] Fava A., Petri M. (2019). Systemic lupus erythematosus: Diagnosis and clinical management. J. Autoimmun..

[2] Tsokos G.C. (2011). Systemic lupus erythematosus. N. Engl. J. Med..

[3] Alduraibi F., Fatima H., Hamilton J.A., Chatham W.W., Hsu H.C., Mountz J.D. (2022). Lupus nephritis correlates with B cell interferon-β, anti-Smith, and anti-DNA: A retrospective study. Arthritis Res. Ther..

[4] Bernatsky S., Boivin J.F., Joseph L., Manzi S., Ginzler E., Gladman D.D., Urowitz M., Fortin P.R., Petri M., Barr S. (2006). Mortality in systemic lupus erythematosus. Arthritis Rheum..

[5] Bernal C.B., Zamora L.D., Navarra S.V. (2015). Biologic therapies in systemic lupus erythematosus. Int. J. Rheum. Dis..

[6] Zhang X., Zhu L., Zhang H., Chen S., Xiao Y. (2022). CAR-T cell therapy in hematological malignancies: Current opportunities and challenges. Front. Immunol..

[7] Lyu X., Gupta L., Tholouli E., Chinoy H. (2024). Chimeric antigen receptor T cell therapy: A new emerging landscape in autoimmune rheumatic diseases. Rheumatology.

[8] Mackensen A., Müller F., Mougiakakos D., Böltz S., Wilhelm A., Aigner M., Völkl S., Simon D., Kleyer A., Munoz L. (2022). Anti-CD19 CAR T cell therapy for refractory systemic lupus erythematosus. Nat. Med..

[9] Mougiakakos D., Krönke G., Völkl S., Kretschmann S., Aigner M., Kharboutli S., Böltz S., Manger B., Mackensen A., Schett G. (2021). CD19-targeted CAR T cells in refractory systemic lupus erythematosus. N. Engl. J. Med..

[10] Mueller F., Taubmann J., Voelkl S., Bucci L., Bergmann C., Aigner M., Wilhelm A., Rothe T., Minopoulou I., Knitza J. (2023). CD19-targeted CAR-T cells in refractory systemic autoimmune diseases: A monocentric experience from the first fifteen patients. Blood.

[11] Taubmann J., Müller F., Boeltz S., Völkl S., Aigner M., Kleyer A., Minnopoulou I., Locatelli F., D’Agostino M.A., Gary R. (2023). OP0141 long term safety and efficacy of car-t cell treatment in refractory systemic lupus erythematosus—Data from the first seven patients. Ann. Rheum. Dis..

[12] Taubmann J., Müller F., Mutlu M.Y., Völkl S., Aigner M., Bozec A., Mackensen A., Grieshaber-Bouyer R., Schett G. (2024). CD19 chimeric antigen receptor T cell treatment: Unraveling the role of b cells in systemic lupus erythematosus. Arthritis Rheumatol..

[13] Oh S., Payne A.S. (2022). Engineering cell therapies for autoimmune diseases: From preclinical to clinical proof of concept. Immune Netw..

[14] Nemazee D. (2017). Mechanisms of central tolerance for B cells. Nat. Rev. Immunol..

[15] Kaminski D.A., Wei C., Qian Y., Rosenberg A.F., Sanz I. (2012). Advances in human B cell phenotypic profiling. Front. Immunol..

[16] Nashi E., Wang Y., Diamond B. (2010). The role of B cells in lupus pathogenesis. Int. J. Biochem. Cell Biol..

[17] Shlomchik M.J., Madaio M.P., Ni D., Trounstein M., Huszar D. (1994). The role of B cells in lpr/lpr-induced autoimmunity. J. Exp. Med..

[18] Furie R., Petri M., Zamani O., Cervera R., Wallace D.J., Tegzová D., Sanchez-Guerrero J., Schwarting A., Merrill J.T., Chatham W.W. (2011). A phase III, randomized, placebo-controlled study of belimumab, a monoclonal antibody that inhibits B lymphocyte stimulator, in patients with systemic lupus erythematosus. Arthritis Rheum..

[19] Furie R.A., Aroca G., Cascino M.D., Garg J.P., Rovin B.H., Alvarez A., Fragoso-Loyo H., Zuta-Santillan E., Schindler T., Brunetta P. (2022). B-cell depletion with obinutuzumab for the treatment of proliferative lupus nephritis: A randomised, double-blind, placebo-controlled trial. Ann. Rheum. Dis..

[20] Weiner G.J. (2010). Rituximab: Mechanism of action. Semin. Hematol..

[21] Forsthuber T.G., Cimbora D.M., Ratchford J.N., Katz E., Stüve O. (2018). B cell-based therapies in CNS autoimmunity: Differentiating CD19 and CD20 as therapeutic targets. Ther. Adv. Neurol. Disord..

[22] Thurlings R.M., Teng O., Vos K., Gerlag D.M., Aarden L., Stapel S.O., Van Laar J.M., Tak P.P., Wolbink G.J. (2010). Clinical response, pharmacokinetics, development of human anti-chimaeric antibodies, and synovial tissue response to rituximab treatment in patients with rheumatoid arthritis. Ann. Rheum. Dis..

[23] Anolik J.H., Barnard J., Owen T., Zheng B., Kemshetti S., Looney R.J., Sanz I. (2007). Delayed memory B cell recovery in peripheral blood and lymphoid tissue in systemic lupus erythematosus after B cell depletion therapy. Arthritis Rheum..

[24] Kamburova E.G., Koenen H.J., Borgman K.J., Ten Berge I.J., Joosten I., Hilbrands L.B. (2013). A single dose of rituximab does not deplete B cells in secondary lymphoid organs but alters phenotype and function. Am. J. Transplant..

[25] Merrill J.T., Neuwelt C.M., Wallace D.J., Shanahan J.C., Latinis K.M., Oates J.C., Utset T.O., Gordon C., Isenberg D.A., Hsieh H.J. (2010). Efficacy and safety of rituximab in moderately-to-severely active systemic lupus erythematosus: The randomized, double-blind, phase II/III systemic lupus erythematosus evaluation of rituximab trial. Arthritis Rheum..

[26] Rovin B.H., Furie R., Latinis K., Looney R.J., Fervenza F.C., Sanchez-Guerrero J., Maciuca R., Zhang D., Garg J.P., Brunetta P. (2012). Efficacy and safety of rituximab in patients with active proliferative lupus nephritis: The lupus nephritis assessment with rituximab study. Arthritis Rheum..

[27] Mysler E.F., Spindler A.J., Guzman R., Bijl M., Jayne D., Furie R.A., Houssiau F.A., Drappa J., Close D., Maciuca R. (2013). Efficacy and safety of ocrelizumab in active proliferative lupus nephritis: Results from a randomized, double-blind, phase III study. Arthritis Rheum..

[28] Arnold J., Dass S., Twigg S., Jones C.H., Rhodes B., Hewins P., Chakravorty M., Courtney P., Ehrenstein M., Yusof M.Y.M. (2022). Efficacy and safety of obinutuzumab in systemic lupus erythematosus patients with secondary non-response to rituximab. Rheumatology.

[29] Dörner T., Kaufmann J., Wegener W.A., Teoh N., Goldenberg D.M., Burmester G.R. (2006). Initial clinical trial of epratuzumab (humanized anti-CD22 antibody) for immunotherapy of systemic lupus erythematosus. Arthritis Res. Ther..

[30] Clowse M.E., Wallace D.J., Furie R.A., Petri M.A., Pike M.C., Leszczyński P., Neuwelt C.M., Hobbs K., Keiserman M., Duca L. (2017). Efficacy and safety of epratuzumab in moderately to severely active systemic lupus erythematosus: Results from two phase III randomized, double-blind, placebo-controlled trials. Arthritis Rheumatol..

[31] Dubey A.K., Handu S.S., Dubey S., Sharma P., Sharma K.K., Ahmed Q.M. (2011). Belimumab: First targeted biological treatment for systemic lupus erythematosus. J. Pharmacol. Pharmacother..

[32] Wallace D.J., Stohl W., Furie R.A., Lisse J.R., McKay J.D., Merrill J.T., Petri M.A., Ginzler E.M., Chatham W.W., McCune W.J. (2009). A phase II, randomized, double-blind, placebo-controlled, dose-ranging study of belimumab in patients with active systemic lupus erythematosus. Arthritis Rheum..

[33] Manzi S., Sánchez-Guerrero J., Merrill J.T., Furie R., Gladman D., Navarra S.V., Ginzler E.M., D’Cruz D.P., Doria A., Cooper S. (2012). Effects of belimumab, a B lymphocyte stimulator-specific inhibitor, on disease activity across multiple organ domains in patients with systemic lupus erythematosus: Combined results from two phase III trials. Ann. Rheum. Dis..

[34] Furie R., Rovin B.H., Houssiau F., Malvar A., Teng Y.K.O., Contreras G., Amoura Z., Yu X., Mok C.C., Santiago M.B. (2020). Two-year, randomized, controlled trial of belimumab in lupus nephritis. N. Engl. J. Med..

[35] Merrill J.T., Van Vollenhoven R.F., Buyon J.P., Furie R.A., Stohl W., Morgan-Cox M., Dickson C., Anderson P.W., Lee C., Berclaz P.Y. (2016). Efficacy and safety of subcutaneous tabalumab, a monoclonal antibody to B-cell activating factor, in patients with systemic lupus erythematosus: Results from ILLUMINATE-2, a 52-week, phase III, multicentre, randomised, double-blind, placebo-controlled study. Ann. Rheum. Dis..

[36] Furie R.A., Leon G., Thomas M., Petri M.A., Chu A.D., Hislop C., Martin R.S., Scheinberg M.A. (2015). A phase 2, randomised, placebo-controlled clinical trial of blisibimod, an inhibitor of B cell activating factor, in patients with moderate-to-severe systemic lupus erythematosus, the PEARL-SC study. Ann. Rheum. Dis..

[37] Merrill J.T., Shanahan W.R., Scheinberg M., Kalunian K.C., Wofsy D., Martin R.S. (2018). Phase III trial results with blisibimod, a selective inhibitor of B-cell activating factor, in subjects with systemic lupus erythematosus (SLE): Results from a randomised, double-blind, placebo-controlled trial. Ann. Rheum. Dis..

[38] Petri M.A., Martin R.S., Scheinberg M.A., Furie R.A. (2017). Assessments of fatigue and disease activity in patients with systemic lupus erythematosus enrolled in the phase 2 clinical trial with blisibimod. Lupus.

[39] Isenberg D., Gordon C., Licu D., Copt S., Rossi C.P., Wofsy D. (2015). Efficacy and safety of atacicept for prevention of flares in patients with moderate-to-severe systemic lupus erythematosus (SLE): 52-week data (APRIL-SLE randomised trial). Ann. Rheum. Dis..

[40] Merrill J.T., Wallace D.J., Wax S., Kao A., Fraser P.A., Chang P., Isenberg D. (2018). Efficacy and safety of atacicept in patients with systemic lupus erythematosus: Results of a twenty-four-week, multicenter, randomized, double-blind, placebo-controlled, parallel-arm, phase IIb study. Arthritis Rheumatol..

[41] Wallace D.J., Isenberg D.A., Morand E.F., Vazquez-Mateo C., Kao A.H., Aydemir A., Pudota K., Ona V., Aranow C., Merrill J.T. (2021). Safety and clinical activity of atacicept in the long-term extension of the phase 2b ADDRESS II study in systemic lupus erythematosus. Rheumatology.

[42] Radic M., Neeli I., Marion T. (2022). Prospects for CAR T cell immunotherapy in autoimmune diseases: Clues from Lupus. Expert Opin. Biol. Ther..

[43] Jayne D., Passweg J., Marmont A., Farge D., Zhao X., Arnold R., Hiepe F., Lisukov I., Musso M., Ou-Yang J. (2004). Autologous stem cell transplantation for systemic lupus erythematosus. Lupus.

[44] De Buys P., Khanna D., Furst D.E. (2005). Hemopoietic stem cell transplantation in rheumatic diseases—An update. Autoimmun. Rev..

[45] Schett G., Mackensen A., Mougiakakos D. (2023). CAR T-cell therapy in autoimmune diseases. Lancet.

[46] Jayaraman J., Mellody M.P., Hou A.J., Desai R.P., Fung A.W., Pham A.H.T., Chen Y.Y., Zhao W. (2020). CAR-T design: Elements and their synergistic function. EBioMedicine.

[47] Chmielewski M., Hombach A., Heuser C., Adams G.P., Abken H. (2004). T cell activation by antibody-like immunoreceptors: Increase in affinity of the single-chain fragment domain above threshold does not increase T cell activation against antigen-positive target cells but decreases selectivity. J. Immunol..

[48] Liu X., Jiang S., Fang C., Yang S., Olalere D., Pequignot E.C., Cogdill A.P., Li N., Ramones M., Granda B. (2015). Affinity-tuned ErbB2 or EGFR chimeric antigen receptor T cells exhibit an increased therapeutic index against tumors in mice. Cancer Res..

[49] Dejenie T.A., Tiruneh G.M.M., Terefe G.D., Admasu F.T., Tesega W.W., Abebe E.C. (2022). Current updates on generations, approvals, and clinical trials of CAR T-cell therapy. Hum. Vaccin. Immunother..

[50] Brocker T. (2000). Chimeric Fv-zeta or Fv-epsilon receptors are not sufficient to induce activation or cytokine production in peripheral T cells. Blood.

[51] Eshhar Z., Waks T., Gross G., Schindler D.G. (1993). Specific activation and targeting of cytotoxic lymphocytes through chimeric single chains consisting of antibody-binding domains and the gamma or zeta subunits of the immunoglobulin and T-cell receptors. Proc. Natl. Acad. Sci. USA.

[52] Acuto O., Michel F. (2003). CD28-mediated co-stimulation: A quantitative support for TCR signalling. Nat. Rev. Immunol..

[53] Hombach A., Wieczarkowiecz A., Marquardt T., Heuser C., Usai L., Pohl C., Seliger B., Abken H. (2001). Tumor-specific T cell activation by recombinant immunoreceptors: CD3 zeta signaling and CD28 costimulation are simultaneously required for efficient IL-2 secretion and can be integrated into one combined CD28/CD3 zeta signaling receptor molecule. J. Immunol..

[54] Imai C., Mihara K., Andreansky M., Nicholson I.C., Pui C.H., Geiger T.L., Campana D. (2004). Chimeric receptors with 4-1BB signaling capacity provoke potent cytotoxicity against acute lymphoblastic leukemia. Leukemia.

[55] Jenkins M.K., Burrell E., Ashwell J.D. (1990). Antigen presentation by resting B cells. Effectiveness at inducing T cell proliferation is determined by costimulatory signals, not T cell receptor occupancy. J. Immunol..

[56] Maher J., Brentjens R.J., Gunset G., Rivière I., Sadelain M. (2002). Human T-lymphocyte cytotoxicity and proliferation directed by a single chimeric TCRzeta /CD28 receptor. Nat. Biotechnol..

[57] Ramos C.A., Rouce R., Robertson C.S., Reyna A., Narala N., Vyas G., Mehta B., Zhang H., Dakhova O., Carrum G. (2018). In vivo fate and activity of second- versus third-generation CD19-specific CAR-T cells in B cell non-Hodgkin’s lymphomas. Mol. Ther..

[58] Sadelain M., Brentjens R., Rivière I. (2013). The basic principles of chimeric antigen receptor design. Cancer Discov..

[59] Schubert M.L., Schmitt A., Neuber B., Hückelhoven-Krauss A., Kunz A., Wang L., Gern U., Michels B., Sellner L., Hofmann S. (2019). Third-generation CAR T cells targeting CD19 are associated with an excellent safety profile and might improve persistence of CAR T cells in treated patients. Blood.

[60] Chmielewski M., Abken H. (2015). TRUCKs: The fourth generation of CARs. Expert Opin. Biol. Ther..

[61] Vormittag P., Gunn R., Ghorashian S., Veraitch F.S. (2018). A guide to manufacturing CAR T cell therapies. Curr. Opin. Biotechnol..

[62] Liu Y., An L., Huang R., Xiong J., Yang H., Wang X., Zhang X. (2022). Strategies to enhance CAR-T persistence. Biomark. Res..

[63] Benjamin R., Graham C., Yallop D., Jozwik A., Mirci-Danicar O.C., Lucchini G., Pinner D., Jain N., Kantarjian H., Boissel N. (2020). Genome-edited, donor-derived allogeneic anti-CD19 chimeric antigen receptor T cells in paediatric and adult B-cell acute lymphoblastic leukaemia: Results of two phase 1 studies. Lancet.

[64] Chong E.A., Ruella M., Schuster S.J. (2021). Five-year outcomes for refractory B-cell lymphomas with CAR T-cell therapy. N. Engl. J. Med..

[65] DiNofia A.M., Grupp S.A. (2021). Will allogeneic CAR T cells for CD19^+^ malignancies take autologous CAR T cells ‘off the shelf’?. Nat. Rev. Clin. Oncol..

[66] Schuster S.J., Svoboda J., Chong E.A., Nasta S.D., Mato A.R., Anak Ö., Brogdon J.L., Pruteanu-Malinici I., Bhoj V., Landsburg D. (2017). Chimeric antigen receptor T cells in refractory B-cell lymphomas. N. Engl. J. Med..

[67] Levine B.L., Miskin J., Wonnacott K., Keir C. (2017). Global manufacturing of CAR T cell therapy. Mol. Ther. Methods Clin. Dev..

[68] Lee D.W., Gardner R., Porter D.L., Louis C.U., Ahmed N., Jensen M., Grupp S.A., Mackall C.L. (2014). Current concepts in the diagnosis and management of cytokine release syndrome. Blood.

[69] Wang X., Rivière I. (2016). Clinical manufacturing of CAR T cells: Foundation of a promising therapy. Mol. Ther. Oncolytics.

[70] Poorebrahim M., Sadeghi S., Fakhr E., Abazari M.F., Poortahmasebi V., Kheirollahi A., Askari H., Rajabzadeh A., Rastegarpanah M., Linē A. (2019). Production of CAR T-cells by GMP-grade lentiviral vectors: Latest advances and future prospects. Crit. Rev. Clin. Lab. Sci..

[71] Akhavan D., Alizadeh D., Wang D., Weist M.R., Shepphird J.K., Brown C.E. (2019). CAR T cells for brain tumors: Lessons learned and road ahead. Immunol. Rev..

[72] Kambayana G., Rini S.S. (2023). Autologous CD19-targeted chimeric antigen receptor (CAR)T-cells as the future of systemic lupus erythematosus treatment. Curr. Rheumatol. Rev..

[73] Fraietta J.A., Lacey S.F., Orlando E.J., Pruteanu-Malinici I., Gohil M., Lundh S., Boesteanu A.C., Wang Y., O’Connor R.S., Hwang W.T. (2018). Determinants of response and resistance to CD19 chimeric antigen receptor (CAR) T cell therapy of chronic lymphocytic leukemia. Nat. Med..

[74] Ghassemi S., Nunez-Cruz S., O’Connor R.S., Fraietta J.A., Patel P.R., Scholler J., Barrett D.M., Lundh S.M., Davis M.M., Bedoya F. (2018). Reducing ex vivo culture improves the antileukemic activity of chimeric antigen receptor (CAR) T cells. Cancer Immunol. Res..

[75] Melenhorst J.J., Chen G.M., Wang M., Porter D.L., Chen C., Collins M.A., Gao P., Bandyopadhyay S., Sun H., Zhao Z. (2022). Decade-long leukaemia remissions with persistence of CD4^+^ CAR T cells. Nature.

[76] Müller F., Taubmann J., Bucci L., Wilhelm A., Bergmann C., Völkl S., Aigner M., Rothe T., Minopoulou I., Tur C. (2024). CD19 CAR T-cell therapy in autoimmune disease—A case series with follow-up. N. Engl. J. Med..

[77] Wang W., He S., Zhang W., Zhang H., DeStefano V.M., Wada M., Pinz K., Deener G., Shah D., Hagag N. BCMA-CD19 compound CAR T cells for systemic lupus erythematosus: A phase 1 open-label clinical trial. Ann. Rheum. Dis..

[78] Hernández J.C., Barba P., Alberich M.L., Fischer O., Kovacs B., Calzascia T., Pearson D., Garrotte A.L.J., Kirsilae T., Siegel R. (2023). An open-label, multi-center, phase 1/2 study to assess safety, efficacy and cellular kinetics of YTB323, a rapid manufacturing CAR-T cell therapy targeting CD19 on B cells, for severe refractory systemic lupus erythematosus: Preliminary results. Arthritis Rheumatol..

[79] Krickau T., Naumann-Bartsch N., Aigner M., Kharboutli S., Kretschmann S., Spoerl S., Vasova I., Völkl S., Woelfle J., Mackensen A. (2024). CAR T-cell therapy rescues adolescent with rapidly progressive lupus nephritis from haemodialysis. Lancet.

[80] Podoll A., Furie R., Kim F., Chou J., Sengupta R., Bayer R., Gutman J., Chung J. (2024). First two US patients with lupus nephritis (LN) treated with anti-CD19 chimeric antigen receptor (CAR) T-cell therapy: Preliminary results from the KYSA-1 phase 1, multicenter study of KYV-101. Lupus Sci. Med..

[81] Marasco E., Bracaglia C., Merli P., Alvarez P., Nicolai R., Algeri M., Cefalo M., Becilli M., Benedetti F., Locatelli F. (2024). Anti-CD19 CAR-T cell therapy for refractory childhood-onset systemic lupus erythematosus. Lupus Sci. Med..

[82] European Alliance of Associations for Rheumatology Welcome to EULAR’s Abstract Archives. https://scientific.sparx-ip.net/archiveeular/?c=s&view=1&searchfor.

[83] Ahuja A., Shupe J., Dunn R., Kashgarian M., Kehry M.R., Shlomchik M.J. (2007). Depletion of B cells in murine lupus: Efficacy and resistance. J. Immunol..

[84] Bekar K.W., Owen T., Dunn R., Ichikawa T., Wang W., Wang R., Barnard J., Brady S., Nevarez S., Goldman B.I. (2010). Prolonged effects of short-term anti-CD20 B cell depletion therapy in murine systemic lupus erythematosus. Arthritis Rheum..

[85] Kansal R., Richardson N., Neeli I., Khawaja S., Chamberlain D., Ghani M., Ghani Q.U., Balazs L., Beranova-Giorgianni S., Giorgianni F. (2019). Sustained B cell depletion by CD19-targeted CAR T cells is a highly effective treatment for murine lupus. Sci. Transl. Med..

[86] Porter D.L., Hwang W.T., Frey N.V., Lacey S.F., Shaw P.A., Loren A.W., Bagg A., Marcucci K.T., Shen A., Gonzalez V. (2015). Chimeric antigen receptor T cells persist and induce sustained remissions in relapsed refractory chronic lymphocytic leukemia. Sci. Transl. Med..

[87] Gardner R.A., Finney O., Annesley C., Brakke H., Summers C., Leger K., Bleakley M., Brown C., Mgebroff S., Kelly-Spratt K.S. (2017). Intent-to-treat leukemia remission by CD19 CAR T cells of defined formulation and dose in children and young adults. Blood.

[88] Dickinson M.J., Barba P., Jäger U., Shah N.N., Blaise D., Briones J., Shune L., Boissel N., Bondanza A., Mariconti L. (2023). A novel autologous CAR-T therapy, YTB323, with preserved T-cell stemness shows enhanced CAR T-cell efficacy in preclinical and early clinical development. Cancer Discov..

[89] Maude S.L., Barrett D., Teachey D.T., Grupp S.A. (2014). Managing cytokine release syndrome associated with novel T cell-engaging therapies. Cancer J..

[90] Hay K.A., Hanafi L.A., Li D., Gust J., Liles W.C., Wurfel M.M., López J.A., Chen J., Chung D., Harju-Baker S. (2017). Kinetics and biomarkers of severe cytokine release syndrome after CD19 chimeric antigen receptor-modified T-cell therapy. Blood.

[91] Maude S.L., Frey N., Shaw P.A., Aplenc R., Barrett D.M., Bunin N.J., Chew A., Gonzalez V.E., Zheng Z., Lacey S.F. (2014). Chimeric antigen receptor T cells for sustained remissions in leukemia. N. Engl. J. Med..

[92] Grupp S.A., Kalos M., Barrett D., Aplenc R., Porter D.L., Rheingold S.R., Teachey D.T., Chew A., Hauck B., Wright J.F. (2013). Chimeric antigen receptor-modified T cells for acute lymphoid leukemia. N. Engl. J. Med..

[93] Davila M.L., Riviere I., Wang X., Bartido S., Park J., Curran K., Chung S.S., Stefanski J., Borquez-Ojeda O., Olszewska M. (2014). Efficacy and toxicity management of 19-28z CAR T cell therapy in B cell acute lymphoblastic leukemia. Sci. Transl. Med..

[94] Zhao J.L., Ma C., O’Connell R.M., Mehta A., DiLoreto R., Heath J.R., Baltimore D. (2014). Conversion of danger signals into cytokine signals by hematopoietic stem and progenitor cells for regulation of stress-induced hematopoiesis. Cell Stem Cell.

[95] Feng X., Scheinberg P., Wu C.O., Samsel L., Nunez O., Prince C., Ganetzky R.D., McCoy J.P., Maciejewski J.P., Young N.S. (2011). Cytokine signature profiles in acquired aplastic anemia and myelodysplastic syndromes. Haematologica.

[96] Rodríguez Mdel C., Bernad A., Aracil M. (2004). Interleukin-6 deficiency affects bone marrow stromal precursors, resulting in defective hematopoietic support. Blood.

[97] Tie R., Li H., Cai S., Liang Z., Shan W., Wang B., Tan Y., Zheng W., Huang H. (2019). Interleukin-6 signaling regulates hematopoietic stem cell emergence. Exp. Mol. Med..

[98] Damoulis P.D., Hauschka P.V. (1997). Nitric oxide acts in conjunction with proinflammatory cytokines to promote cell death in osteoblasts. J. Bone Miner. Res..

[99] Saini N.K., Sinha R., Singh P., Sharma M., Pathak R., Rathor N., Varma-Basil M., Bose M. (2016). Mce4A protein of *Mycobacterium tuberculosis* induces pro inflammatory cytokine response leading to macrophage apoptosis in a TNF-α dependent manner. Microb. Pathog..

[100] Diorio C., Shraim R., Myers R., Behrens E.M., Canna S., Bassiri H., Aplenc R., Burudpakdee C., Chen F., DiNofia A.M. (2022). Comprehensive serum proteome profiling of cytokine release syndrome and immune effector cell-associated neurotoxicity syndrome patients with B-cell all receiving CAR T19. Clin. Cancer Res..

[101] Buechner J., Grupp S.A., Hiramatsu H., Teachey D.T., Rives S., Laetsch T.W., Yanik G.A., Wood P., Awasthi R., Yi L. (2021). Practical guidelines for monitoring and management of coagulopathy following tisagenlecleucel CAR T-cell therapy. Blood Adv..

[102] Zhou J., Zhang Y., Shan M., Zong X., Geng H., Li J., Chen G., Yu L., Xu Y., Li C. (2022). Cytopenia after chimeric antigen receptor T cell immunotherapy in relapsed or refractory lymphoma. Front. Immunol..

[103] Wang L., Hong R., Zhou L., Wang Y., Lv Y., Ni F., Zhang M., Zhao H., Ding S., Chang A.H. (2023). Cytokine profiles are associated with prolonged hematologic toxicities after B-cell maturation antigen targeted chimeric antigen receptor-T-cell therapy. Cytotherapy.

[104] Qi K., Yan Z., Cheng H., Chen W., Wang Y., Wang X., Cao J., Zhang H., Sang W., Zhu F. (2021). An analysis of cardiac disorders associated with chimeric antigen receptor T cell therapy in 126 patients: A single-centre retrospective study. Front. Oncol..

[105] Brentjens R., Yeh R., Bernal Y., Riviere I., Sadelain M. (2010). Treatment of chronic lymphocytic leukemia with genetically targeted autologous T cells: Case report of an unforeseen adverse event in a phase I clinical trial. Mol. Ther..

[106] Porter D.L., Levine B.L., Kalos M., Bagg A., June C.H. (2011). Chimeric antigen receptor-modified T cells in chronic lymphoid leukemia. N. Engl. J. Med..

[107] Kochenderfer J.N., Dudley M.E., Feldman S.A., Wilson W.H., Spaner D.E., Maric I., Stetler-Stevenson M., Phan G.Q., Hughes M.S., Sherry R.M. (2012). B-cell depletion and remissions of malignancy along with cytokine-associated toxicity in a clinical trial of anti-CD19 chimeric-antigen-receptor-transduced T cells. Blood.

[108] Kalos M., Levine B.L., Porter D.L., Katz S., Grupp S.A., Bagg A., June C.H. (2011). T cells with chimeric antigen receptors have potent antitumor effects and can establish memory in patients with advanced leukemia. Sci. Transl. Med..

[109] Pepys M.B., Hirschfield G.M. (2003). C-reactive protein: A critical update. J. Clin. Investig..

[110] U.S. Department of Health and Human Services (2017). Common Terminology Criteria for Adverse Events (CTCAE) Version 5.0. https://ctep.cancer.gov/protocoldevelopment/electronic_applications/docs/CTCAE_v5_Quick_Reference_5×7.pdf.

[111] Mitchell C.D., Richards S.M., Kinsey S.E., Lilleyman J., Vora A., Eden T.O. (2005). Benefit of dexamethasone compared with prednisolone for childhood acute lymphoblastic leukaemia: Results of the UK medical research council ALL97 randomized trial. Br. J. Haematol..

[112] Teachey D.T., Rheingold S.R., Maude S.L., Zugmaier G., Barrett D.M., Seif A.E., Nichols K.E., Suppa E.K., Kalos M., Berg R.A. (2013). Cytokine release syndrome after blinatumomab treatment related to abnormal macrophage activation and ameliorated with cytokine-directed therapy. Blood.

[113] Alten R. (2011). Tocilizumab: A novel humanized anti-interleukin 6 receptor antibody for the treatment of patients with rheumatoid arthritis. Ther. Adv. Musculoskelet. Dis..

[114] Flammiger A., Fiedler W., Bacher U., Bokemeyer C., Schneider M., Binder M. (2012). Critical imbalance of TNF-α and soluble TNF receptor 1 in a patient with macrophage activation syndrome: Potential implications for diagnostics and treatment. Acta Haematol..

[115] Si S., Teachey D.T. (2020). Spotlight on tocilizumab in the treatment of CAR-T-cell-induced cytokine release syndrome: Clinical evidence to date. Ther. Clin. Risk Manag..

[116] Gabay C., Lamacchia C., Palmer G. (2010). IL-1 pathways in inflammation and human diseases. Nat. Rev. Rheumatol..

[117] Prahalad S., Bove K.E., Dickens D., Lovell D.J., Grom A.A. (2001). Etanercept in the treatment of macrophage activation syndrome. J. Rheumatol..

[118] Hayden P.J., Roddie C., Bader P., Basak G.W., Bonig H., Bonini C., Chabannon C., Ciceri F., Corbacioglu S., Ellard R. (2022). Management of adults and children receiving CAR T-cell therapy: 2021 best practice recommendations of the European society for blood and marrow transplantation (EBMT) and the joint accreditation committee of ISCT and EBMT (JACIE) and the European haematology association (EHA). Ann. Oncol..

[119] Sheth V.S., Gauthier J. (2021). Taming the beast: CRS and ICANS after CAR T-cell therapy for ALL. Bone Marrow Transplant..

[120] Nellan A., McCully C.M.L., Garcia R.C., Jayaprakash N., Widemann B.C., Lee D.W., Warren K.E. (2018). Improved CNS exposure to tocilizumab after cerebrospinal fluid compared to intravenous administration in rhesus macaques. Blood.

[121] Yakoub-Agha I., Chabannon C., Bader P., Basak G.W., Bonig H., Ciceri F., Corbacioglu S., Duarte R.F., Einsele H., Hudecek M. (2020). Management of adults and children undergoing chimeric antigen receptor T-cell therapy: Best practice recommendations of the European society for blood and marrow transplantation (EBMT) and the joint accreditation committee of ISCT and EBMT (JACIE). Haematologica.

[122] Santomasso B.D., Nastoupil L.J., Adkins S., Lacchetti C., Schneider B.J., Anadkat M., Atkins M.B., Brassil K.J., Caterino J.M., Chau I. (2021). Management of immune-related adverse events in patients treated with chimeric antigen receptor T-cell therapy: ASCO guideline. J. Clin. Oncol..

[123] Yan Z., Cao J., Cheng H., Qiao J., Zhang H., Wang Y., Shi M., Lan J., Fei X., Jin L. (2019). A combination of humanised anti-CD19 and anti-BCMA CAR T cells in patients with relapsed or refractory multiple myeloma: A single-arm, phase 2 trial. Lancet Haematol..

[124] Jain M.D., Smith M., Shah N.N. (2023). How I treat refractory CRS and ICANS after CAR T-cell therapy. Blood.

[125] Sun S., Hao H., Yang G., Zhang Y., Fu Y. (2018). Immunotherapy with CAR-modified T cells: Toxicities and overcoming strategies. J. Immunol. Res..

[126] Zahid U., Shaukat A.A., Hassan N., Anwer F. (2017). Coccidioidomycosis, immunoglobulin deficiency: Safety challenges with CAR T cells therapy for relapsed lymphoma. Immunotherapy.

[127] Howard S.C., Jones D.P., Pui C.H. (2011). The tumor lysis syndrome. N. Engl. J. Med..

[128] Bonifant C.L., Jackson H.J., Brentjens R.J., Curran K.J. (2016). Toxicity and management in CAR T-cell therapy. Mol. Ther. Oncolytics.

[129] Lamers C.H., Sleijfer S., Van Steenbergen S., Van Elzakker P., Van Krimpen B., Groot C., Vulto A., Den Bakker M., Oosterwijk E., Debets R. (2013). Treatment of metastatic renal cell carcinoma with CAIX CAR-engineered T cells: Clinical evaluation and management of on-target toxicity. Mol. Ther..

[130] Morgan R.A., Yang J.C., Kitano M., Dudley M.E., Laurencot C.M., Rosenberg S.A. (2010). Case report of a serious adverse event following the administration of T cells transduced with a chimeric antigen receptor recognizing ERBB2. Mol. Ther..

[131] Hombach A., Hombach A.A., Abken H. (2010). Adoptive immunotherapy with genetically engineered T cells: Modification of the IgG1 Fc ‘spacer’ domain in the extracellular moiety of chimeric antigen receptors avoids ‘off-target’ activation and unintended initiation of an innate immune response. Gene Ther..

[132] Cameron B.J., Gerry A.B., Dukes J., Harper J.V., Kannan V., Bianchi F.C., Grand F., Brewer J.E., Gupta M., Plesa G. (2013). Identification of a titin-derived HLA-A1-presented peptide as a cross-reactive target for engineered MAGE A3-directed T cells. Sci. Transl. Med..

[133] Linette G.P., Stadtmauer E.A., Maus M.V., Rapoport A.P., Levine B.L., Emery L., Litzky L., Bagg A., Carreno B.M., Cimino P.J. (2013). Cardiovascular toxicity and titin cross-reactivity of affinity-enhanced T cells in myeloma and melanoma. Blood.

[134] Curran K.J., Pegram H.J., Brentjens R.J. (2012). Chimeric antigen receptors for T cell immunotherapy: Current understanding and future directions. J. Gene Med..

[135] Lamers C.H., Willemsen R., Van Elzakker P., Van Steenbergen-Langeveld S., Broertjes M., Oosterwijk-Wakka J., Oosterwijk E., Sleijfer S., Debets R., Gratama J.W. (2011). Immune responses to transgene and retroviral vector in patients treated with ex vivo-engineered T cells. Blood.

[136] Maus M.V., Haas A.R., Beatty G.L., Albelda S.M., Levine B.L., Liu X., Zhao Y., Kalos M., June C.H. (2013). T cells expressing chimeric antigen receptors can cause anaphylaxis in humans. Cancer Immunol. Res..

[137] Food and Drug Administration Considerations for the Development of Chimeric Antigen Receptor (CAR) T Cell Products. https://www.fda.gov/regulatory-information/search-fda-guidance-documents/considerations-development-chimeric-antigen-receptor-car-t-cell-products.

[138] Verdun N., Marks P. (2024). Secondary cancers after chimeric antigen receptor T-cell therapy. N. Engl. J. Med..

[139] US Food & Drug Administration FDA Investigating Serious Risk of T-Cell Malignancy Following BCMA-Directed or CD19-Directed Autologous Chimeric Antigen Receptor (CAR) T Cell Immunotherapies. https://www.fda.gov/vaccines-blood-biologics/safety-availability-biologics/fda-investigating-serious-risk-t-cell-malignancy-following-bcma-directed-or-cd19-directed-autologous.

[140] Tokarew N., Ogonek J., Endres S., Von Bergwelt-Baildon M., Kobold S. (2019). Teaching an old dog new tricks: Next-generation CAR T cells. Br. J. Cancer.

[141] Wu L.L. FDA’s Peter Marks Says Some Secondary Cancer Cases after CAR-T Therapy May Be ‘Causal,’ But Benefits Still Outweigh Risks: #JPM24. https://endpts.com/jpm24-fdas-peter-marks-says-some-secondary-cancer-cases-after-car-t-therapy-may-be-causal-but-benefits-still-outweigh-risks/.

[142] US Food & Drug Administration FDA Requires Boxed Warning for T Cell Malignancies Following Treatment with BCMA-Directed or CD19-Directed Autologous Chimeric Antigen Receptor (CAR) T Cell Immunotherapies. https://www.fda.gov/vaccines-blood-biologics/safety-availability-biologics/fda-requires-boxed-warning-t-cell-malignancies-following-treatment-bcma-directed-or-cd19-directed.

[143] Levine B.L., Pasquini M.C., Connolly J.E., Porter D.L., Gustafson M.P., Boelens J.J., Horwitz E.M., Grupp S.A., Maus M.V., Locke F.L. (2024). Unanswered questions following reports of secondary malignancies after CAR-T cell therapy. Nat. Med..

[144] Harrison S.J., Nguyen T., Rahman M., Er J., Li J., Li K., Lendvai N., Schecter J.M., Banerjee A., Roccia T. (2023). CAR^+^ T-cell lymphoma post ciltacabtagene autoleucel therapy for relapsed refractory multiple myeloma. Blood.

[145] European Medicines Agency Pharmacovigilance Risk Assessment Committee (PRAC). https://www.ema.europa.eu/en/committees/pharmacovigilance-risk-assessment-committee-prac.

[146] Bouziana S., Bouzianas D. (2024). The current landscape of secondary malignancies after CAR T-cell therapies: How could malignancies be prevented?. Int. J. Mol. Sci..

[147] US Food & Drug Administration BCMA-Directed or CD19-Directed Autologous Chimeric Antigen Receptor (CAR) T Cell Immunotherapies: FDA Safety Communication—FDA Investigating Serious Risk of T-Cell Malignancy. https://www.fda.gov/safety/medical-product-safety-information/bcma-directed-or-cd19-directed-autologous-chimeric-antigen-receptor-car-t-cell-immunotherapies-fda.

[148] Hu L. Clinical Development of Chimeric Antigen Receptor (CAR)-T Cell Therapy in Cancer. https://www.fda.gov/media/167537/download.

[149] Bansal R., Vergidis P., Tosh P., Wilson J.W., Hathcock M., Bennani N.N., Paludo J., Villasboas J.C., Wang Y., Ansell S.M. (2021). Vaccine titers in lymphoma patients receiving chimeric antigen receptor T-cell therapy. J. Clin. Oncol..

[150] Rahman Z.A., Gannon N., Melody M., Muniz P., Ayala E., Roy V., Brumble L., Ailawadhi S., Sher T., Foran J.M. (2019). Impact of anti-CD19 CAR-T axicabtagene ciloleucel on vaccine titers of DTaP and MMR. Blood.

[151] Shah N., Alarcon A., Palazzo M., Ruiz J.D., Batlevi C.W., Dahi P.B., Palomba M.L., Scordo M., Giralt S.A., Sauter C.S. (2021). High rates of residual vaccine titers at 1-year post CD19 chimeric antigen receptor T cell therapy. Transplant. Cell. Ther..

[152] Walti C.S., Krantz E.M., Maalouf J., Boonyaratanakornkit J., Keane-Candib J., Joncas-Schronce L., Stevens-Ayers T., Dasgupta S., Taylor J.J., Hirayama A.V. (2021). Antibodies against vaccine-preventable infections after CAR-T cell therapy for B cell malignancies. JCI Insight.

[153] Bhoj V.G., Arhontoulis D., Wertheim G., Capobianchi J., Callahan C.A., Ellebrecht C.T., Obstfeld A.E., Lacey S.F., Melenhorst J.J., Nazimuddin F. (2016). Persistence of long-lived plasma cells and humoral immunity in individuals responding to CD19-directed CAR T-cell therapy. Blood.

[154] Walti C.S., Loes A.N., Shuey K., Krantz E.M., Boonyaratanakornkit J., Keane-Candib J., Loeffelholz T., Wolf C.R., Taylor J.J., Gardner R.A. (2021). Humoral immunogenicity of the seasonal influenza vaccine before and after CAR-T-cell therapy: A prospective observational study. J. Immunother. Cancer.

